# Context-sensitivity of isosteric substitutions of non-Watson–Crick basepairs in recurrent RNA 3D motifs

**DOI:** 10.1093/nar/gkab703

**Published:** 2021-08-17

**Authors:** Emil F Khisamutdinov, Blake A Sweeney, Neocles B Leontis

**Affiliations:** Department of Chemistry and Center for Photochemical Science, Bowling Green State University, Bowling Green, OH 43403, USA; Department of Chemistry, Ball State University, Muncie, IN 47306, USA; Department of Biological Sciences, Bowling Green State University, Bowling Green, OH 43403, USA; European Molecular Biology Laboratory, European Bioinformatics Institute, Cambridge, UK; Department of Chemistry and Center for Photochemical Science, Bowling Green State University, Bowling Green, OH 43403, USA

## Abstract

Sequence variation in a widespread, recurrent, structured RNA 3D motif, the Sarcin/Ricin (S/R), was studied to address three related questions: First, how do the stabilities of structured RNA 3D motifs, composed of non-Watson–Crick (non-WC) basepairs, compare to WC-paired helices of similar length and sequence? Second, what are the effects on the stabilities of such motifs of isosteric and non-isosteric base substitutions in the non-WC pairs? And third, is there selection for particular base combinations in non-WC basepairs, depending on the temperature regime to which an organism adapts? A survey of large and small subunit rRNAs from organisms adapted to different temperatures revealed the presence of systematic sequence variations at many non-WC paired sites of S/R motifs. UV melting analysis and enzymatic digestion assays of oligonucleotides containing the motif suggest that more stable motifs tend to be more rigid. We further found that the base substitutions at non-Watson–Crick pairing sites can significantly affect the thermodynamic stabilities of S/R motifs and these effects are highly context specific indicating the importance of base-stacking and base-phosphate interactions on motif stability. This study highlights the significance of non-canonical base pairs and their contributions to modulating the stability and flexibility of RNA molecules.

## INTRODUCTION

Compared to double stranded DNA, the interaction patterns of bases in structured RNA molecules are considerably more complex. Atomic resolution structures of large RNAs have revealed that a large fraction of interacting bases do not form Watson–Crick (WC) base pairs. In fact, the standard *cis* Watson − Crick/Watson–Crick (cWW) AU and GC base pairs only account for ∼70% of all base pairs in structured RNAs including the small and large ribosomal RNA subunits (1–3). The remaining one-third of all base pairs are non-WC base pairs. The non-WC base pairs occur primarily in hairpin, internal or junction loops. They serve to structure these loops and to expose functional groups, especially those on the Watson–Crick edges, to form specific binding sites for proteins, small molecule ligands, metals, or other RNAs (4).

Previous work on RNA adaptation to different environments has focused on the ratio of GC to AU Watson–Crick base pairs (5) as GC base pairs are generally more stable than AU pairs. These authors found that in ribosomal RNA (rRNA) the relationship between the proportion of GC base combinations at Watson–Crick base paired positions and the optimal growth temperatures is very weak up to ∼60°C optimal growth temperature. Above this temperature, there is a roughly linear relationship between the percent of GC pairs and the optimal temperature, as GC base pairs generally confer more stability to Watson–Crick helices than AU pairs. The same positive correlation of GC content with optimal growth temperature was found in helices of the RNA component of the signal recognition particle among Archaea (6). In contrast, no such simple relationship was found to exist for genomic DNA; the average GC content varies widely from approximately 30% to more than 60% in bacteria irrespective of the optimal growth temperature (7). Many loop regions are important for the function and overall structure of RNA molecules. Therefore, we suspect that the sequences of loop regions may also adapt to temperature.

In our recent work, we compared the 3D structures of the rRNAs of the distantly related bacteria *Escherichia coli* (mesophile) and *Thermus thermophilus* (thermophile) by aligning the structurally conserved base-paired positions in the 5S, 16S and 23S rRNAs (1). We aligned all non-WC as well as Watson–Crick base pairs, and found that (i) the geometric families of aligned base pairs were nearly 100% conserved, and (ii) almost all base substitutions resulted in isosteric or near isosteric base pairs. This result strongly supported the hypothesis that base pair isostericity is fundamental for understanding the rules of sequence adaptation during RNA evolution. Isostericity indicates which base pairs can potentially substitute for each other preserving the 3D structure of the motif. However, nothing is known about how the isosteric base substitutions impact the stabilities of well-structured RNA 3D motifs and, if there is a selection for particular base combinations depending on optimal growth temperature of the organism. These related questions were addressed in this paper.

The energetic values or RNA base pairs have been studied for several decades. Initially, the focus was on the canonical (i.e., G-C, A-U, and G-U) base pairs and later studies were included non-canonical base pairs (8–11). Particularly, thermodynamic studies of RNA internal loops have taken a ‘bottom up’ approach which attempts to cover the sequence space of internal loops starting systematically with small loops (1 × 1, 1 × 2, 2 × 2, 1 × 3 and 2 × 3) (12). Such an approach quickly confronts the problem of combinatorial explosion. It is simply not possible to study all possible sequence variants of internal loops of size 4 × 4 or greater without a high throughput methodology.

In this work, we use a different approach. We chose a recurrent, well-structured and relatively large internal loop (5 × 4 nucleotides not counting the closing bases or 6 × 5 with closing bases) for detailed study. We have chosen the sarcin-ricin (S/R) motif as the focus of study of phylogenetic and ecological sequence variation and thermodynamic measurements for these reasons:

(i) The motif is recurrent and widespread in structured RNA molecules (13–15); (ii) It is highly conserved, yet different sequence compositions can give essentially the same 3D structure of S/R motif as it will be shown below; (iii) the S/R motif is modular and autonomous, it occurs independently in different molecules and in different locations in the same molecule (16); (iv) the motif is unique, the G-bulged region of the S/R motif was found to have unusually complex electronic structure arrangement due to the combination of S-turn, stacking and base-phosphate (BPh) interactions which was not observed in similar small RNA internal loops (17) making the S/R motif very difficult target for molecular modeling and simulation force fields.

We began by cataloguing the sequence variations observed in atomic-resolution 3D structures, and then we examined sequence variations of the motif at corresponding positions in aligned sequences. We collected these data to choose sequence variants for thermodynamic characterization. We also studied sequences containing single mutations that are predicted to disrupt the motif, guided by base pair isostericity matrices (18).

Thermodynamic analysis of S/R motif variations found in organisms occupying different temperature niches and present in 3D structures indicates that a single change in a non-WC pair can significantly affect the overall stability of the motif. To verify whether sequence variants that have different thermodynamic stabilities still form the same motif, we probed their solution structures using single-strand specific nucleases T1 and A, double strand specific nuclease V1, as well as, DEPC. We found that isosteric base pair substitutions preserve the overall conformation of the motif, while single base substitutions that disrupt key non-WC base pairs destabilize the entire S/R motif.

## MATERIALS AND METHODS

### RNA oligonucleotides

*RNA oligonucleotides* used for thermodynamic studies were obtained from IDT DNA (www.idtdna.com) and purified using preparative denaturing polyacrylamide gel electrophoresis (PAGE) or preparative 20 cm × 20 cm thin-layer chromatography (TLC) (*n*-propanol:ammonium hydroxide:water, 55:35:10). RNA was eluted from gel slices or TLC matrix using a buffer containing 0.3 M NaCl, 10 mM Tris pH 8.0, 0.5 mM EDTA, then ethanol precipitated with 2.5 volume of cold 100% ethanol, rinsed twice with cold 80% ethanol, dried, and dissolved in 30 μl of double deionized (dd H_2_O) water. Purified RNAs produced single bands on a 16.5 cm × 20 cm analytical denaturing 20% polyacrylamide gel. For UV-melting measurements, the RNA stock solutions were extensively dialyzed first against 10 mM EDTA, 100 mM NaCl, and 10 mM sodium cacodylate and then against dd H_2_O and dissolved in dd H_2_O for storage. For each melting profile, a small volume of the oligomer stock was dissolved in 0.1 ml of buffer containing 10 mM sodium cacodylate (pH 6.9), 0.5 mM EDTA and either 10 mM MgCl_2_ and 100 mM NaCl or just 1.0 M NaCl.

*The RNA hairpin molecules* for structural probing were prepared by runoff transcription of PCR amplified DNA templates. Synthetic DNA molecules coding for the anti-sense sequence of the desired RNA were purchased from IDT DNA (www.idtdna.com) and amplified by PCR using primers containing the T7 RNA polymerase promoter. Template strands for T7 transcription were designed using the online program written by Jesse Stombaugh (link: http://rna.bgsu.edu/oldwebsite/rnatodna.html). PCR products were purified using the QiaQuick PCR purification kit (Qiagen Sciences, Maryland 20874). RNA molecules were prepared by *in vitro* transcription using T7 RNA polymerase (Takara Bio Inc., http://www.takara-bio.com) and purified on denaturing PAGE as describe above.

#### 5′-end ^32^P ATP labeling of RNA oligonucleotides

RNA hairpins, prepared by *in vitro* transcription, were labeled with gamma ^32^P at the 5′ terminus. Molecules were first dephosphorylated using Calf Intestinal alkaline phosphatase—10 000 U/ml (CIP, http://www.neb.com) using the manufacture's procedure. The dephosphorylated RNAs were 5′-end labeled with [γ-^32^P] ATP (PerkinElmer Co.) using T4 polynucleotide kinase (New England Biolabs Inc.) according to procedure adopted from Ambion Applied Biosystems INC (http://www.ambion.com/techlib/misc/RNA5_labeling.html). Labeled RNAs were purified on 20% denaturing PAGE before use.

### Thermodynamic experiments

#### Optical melting experiments

Absorbance versus temperature profiles were measured to determine the melting transitions of the duplex RNA molecules and to characterize the temperature-dependent transitions of the single strands. To form duplexes, complementary single strands were mixed at equimolar concentrations. All melting curves were obtained in buffer containing 10 mM sodium cacodylate buffer (pH 6.9), 0.5 mM EDTA and either 0.1 M NaCl/10 mM MgCl_2_ or just 1 M NaCl. Single-stranded extinction coefficients were calculated from the extinction coefficients for dinucleotide monophosphate and nucleosides using the literature parameters (19). Strand concentrations were determined from high-temperature (90°C) absorbance at 260 nm (20). Absorbances were measured in 1 cm path length quartz cuvettes (Starna Cells, Inc), using a 100 CARY-BIO UV-Visible spectrophotometer (Varian, Inc.) equipped with a thermoelectrically controlled cell holder. Absorbances were obtained as a function of temperature at 260 nm or 280 nm with a heating rate of 1°C/min. Absorbance readings were taken every 0.5°C over the range 15–90°C. At least five melting transitions were obtained for each sample.

The transitions of RNA duplexes melted in presence of 1 M NaCl were reversible for two temperature cycles and no significant changes in *T*_M_ were observed. However, transitions in the presence of Mg^2+^ were not reversible since Mg^2+^ catalyzes the hydrolysis of RNA backbone (21).

#### Data analysis of UV denaturation curve

Thermodynamic parameters (Δ*G*_37_°, Δ*H*°, Δ*S*°) for duplex formation of RNA S/R motif constructs were calculated from the melting profiles by curve fitting using the MeltWin (version 3.0) software. This program fits the shape of each curve to the two-state model with sloping base lines given by Equation (1) using a nonlinear least-squares procedure (22).

(1) $$\begin{equation*}{{\rm{A}}_{\rm{T}}}{\rm{ = (1 - f) \times }}{{\rm{A}}_{{\rm{SS}}}}{\rm{ + f}}\,{\rm{ \times }}{{\rm{A}}_{{\rm{DS}}}}\end{equation*}$$

In Equation (1): *A*_T_ is the total absorbance, *A*_SS_ is the absorbance of RNA single strands, *A*_DS_ is the absorbance of the RNA double strand complex and *f* is the fraction of total RNA in the duplex form. Assuming the transitions from the duplex to single strands conform to the two-state model, then, the fraction of RNA duplexes can be related to equilibrium dissociation constant *K*_D_ according to Equation (2):

(2) $$\begin{equation*}{{\rm{K}}_{\rm{D}}}{\rm{ = }}{{\rm{C}}_{\rm{T}}}{\rm{ \times (1 - \;f}}{{\rm{)}}^{\rm{2}}}{\rm{/ }}\left( {{\rm{2 \times f}}} \right)\end{equation*}$$

where *C*_T_ is the total RNA concentration. This allows for the extraction of the thermodynamic parameters assuming linear sloping baselines and temperature independent Δ*H*° and ΔS° values (22):

(3) $$\begin{equation*}{\rm{K = exp}}\left[ {{\rm{ - \Delta H^\circ /R + \Delta S^\circ /RT}}} \right]\end{equation*}$$

Correlation coefficients (*R*^2^) of ≥0.99 were observed for the fits to the melting curves using Equation (1). Reported Δ*H*° and Δ*S*° values and their standard deviations were based on at least five separate experiments for each sample.

To confirm the validity of the two-state model for the duplexes we measured *T*_M_ as a function of *C*_T_ for the U2G/C12A variant at four concentrations ranging from 5 to 20 μM. We constructed a Van’t Hoff plot to calculate thermodynamic parameters of non-self complementary duplexes according to the method of Borer *et al.* (23):

(4) $$\begin{equation*}{\rm{1/}}{{\rm{T}}_{\rm{m}}}{\rm{ = }}\left( {{\rm{R/\Delta H^\circ }}} \right){\rm{ \times ln}}\left( {{{\rm{C}}_{\rm{T}}}{\rm{/4}}} \right){\rm{ + \Delta S^\circ /\Delta H^\circ }}\end{equation*}$$

where *R* is the gas constant, 1.987 cal mol^−1^ K^−1^; *T*_m_ is melting temperature of a duplex.

All transitions confirm the two-state model, Δ*H*° values from the two methods generally agree within 10% error (24,25).

The Gibbs free energy change at 37°C was obtained using the following equation:

(5) $$\begin{equation*}{\rm{\Delta G}}{{\rm{^\circ }}_{{\rm{37}}}}{\rm{ = \Delta H^\circ - }}\left( {{\rm{310}}{\rm{.15 K}}} \right){\rm{\Delta S^\circ }}\end{equation*}$$

### RNA structure probing

#### Limited enzymatic digestion of labeled hairpins

Prior to digestions, samples of radiolabeled hairpins 1 μl (1 μl, ∼2 pmol = 100 000 cpm) containing 1 μl yeast tRNA (10 mg/ml) were heated to 94°C in 5 μl of dd H_2_O for 2 min (denaturing step) and snap-cooled on ice. To this we added 1 μl of 10 **×** Tris–HCl buffer (1 M Tris pH 8.2, 2 M KCl, 10 mM EDTA) and the resulting mixtures were incubated at 30°C for 10 min (annealing step). Finally, 1 μl of 50 mM MgCl_2_ was added and the mixture incubation continued for 30 min (assembling step). RNA samples were digested using 1 μl of T1 (1 U/μl), or A (0.011 ng/μl) or V1 (0.0005 U/μl) for 1 min at 30°C. The digestion reactions were stopped by adding 10 μl of colorless gel-loading solution (10 M urea, 1.5 mM EDTA). The products of digestions were analyzed on 8 M urea–15% sequencing PAGE.

#### Diethylpyrocarbonate modification and cleavage of RNA hairpin bases

RNA chemical modification by a diethylpyrocarbonate (DEPC) was performed under native conditions. Briefly, labeled RNA hairpins (1 μl, ∼ 100 000 cpm) were annealed in SC buffer (pH 6.9) in presence of 10 μg yeast tRNA. Next, 15 μl of DEPC (SigmaAldrich) was added to each RNA sample. Samples were incubated for 30 min at 20°C with mixing every 10 min. Treated RNA strands were precipitated with 2.5 volume of cold ethanol and dried RNA pellets were resuspended in 20 μl aniline-acetate buffer (1 M Aniline, 13.9 N glacial acetic acid, pH 4.5). The cleavage reaction was performed by incubating the mixture for 15 min at 60°C in the dark. Samples were frozen on dry ice and then lyophilized in the SpeedVac. Pellets were resuspended in 50 μl of dd H_2_O, frozen, and dried again on the SpeedVac to remove organics. Finally, cleaved RNA products were resuspended in 6 μl of denaturing loading dye solution (8 M urea, Tris–HCl pH 7.4, EDTA 1 mM, 0.1% (w/v) bromophenol blue) and analyzed on 8 M urea–15% sequencing PAGE.

## RESULTS AND DISCUSSION

### Searching for sarcin-ricin motifs in the 3D structure database

**Figure 1.**
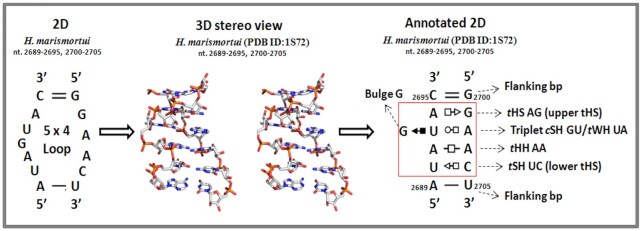
Different representations of the S/R motif from helix- 95 of *H. marismortui* 23S rRNA. Left: 2D structure of 5 × 4 nt asymmetric internal loop, 3D stereo view from PDB ID 1S72 showing non-canonical base pairs. Right: 2D diagram with annotation of all five non-canonical interactions in structure of S/R motif.

Like many other ‘loops’ the nucleotides of S/R motif are represented as single-stranded in 2D structure. The standard S/R motif is an asymmetrical 5 × 4 internal loop (Figure 1, left), although some versions comprise two more nucleotides (6 × 5 internal loop). The 3D structure (Figure 1, center) shows that all of bases are, in fact, involved in non-WC base pairing, resulting in 5 bp and one base triple. The essential features of the 3D structure are depicted using symobls from the Leontis-Westhof base pair classification (Figure 1, right) (4).

To examine the sequence variation in the S/R motifs present in atomic resolution 3D structures, we carried out mixed symbolic and geometric searches, using WebFR3D (26). We searched a non-redundant set of annotated RNA 3D structures from the PDB with resolution better than 4 Å. (http://rna.bgsu.edu/rna3dhub/nrlist) using geometric discrepancy 0.8 Å/nucleotide as the similarity cutoff (27). The non-redundant set contained ribosomal structures from six different organisms. Most notably, the existing data allowed us to compare sequence variation between two related microbial organisms, the mesophile *Deinococcus radiodurans* and the thermophile *T. thermophilus*, that have adapted to different thermal environments, as well as between two phylogenetically diverged organisms that have adapted to similar thermal ranges, the mesophiles *E. coli* and *D. radiodurans*.

**Figure 2.**
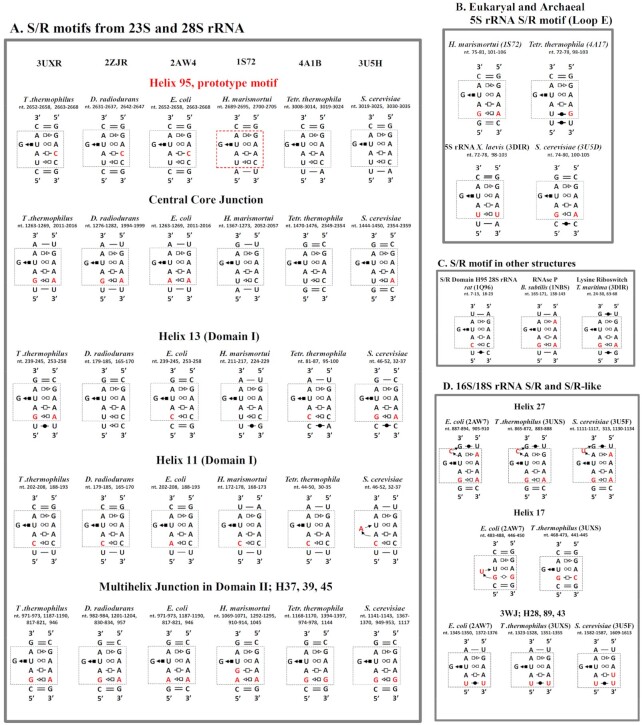
2D diagrams of S/R motif from different organisms found in 3D rRNA data base. (**A**) S/R motifs from large ribosomal subunit, the pdb code of the structures is given on the top. Rows represent diagrams of S/R motif corresponding to particular place in the rRNA and name with the nucleotide numbers are given on the top of each diagram. Each column is structure of particular organism. (**B**) S/R motif diagrams from archaea and eukaryal 5S rRNA, pdb codes are given in parentheses. (**C**) S/R motif diagram from other structures. (**D**) S/R-like motifs from 16S rRNA 3D structures of *E. coli* and *T. thermophilus* and 18S rRNA of *S. cerevisiae*.

Figure 2 shows 45 instances of S/R and S/R-like motifs that have been identified in 15 distinct PDB files (listed in Table 1). Consistent with previous results (18), five recurrent S/R motifs are found in conserved positions in the large ribosomal subunit (LSU) of each of the six organisms and three in the small ribosomal unit (SSU). Throughout this paper we will refer to the S/R motif located in H95 of 23S rRNA from *Haloarcula marismortui* as the prototype, the sequence of which is indicated by the red rectangle in the upper row of Figure 2A. The H95 motif of bacterial 23S rRNA is sensitive to the toxins sarcin and ricin giving the motif its name (28).

**Table 1.** Representative PDB structure files containing S/R motifs

Organisms	Temperature preference	RNA type	PDB ID	References
**Archaeon**				
*H. marismortui*	Mesophile	50S (LSU)	1S72	(49)
**Bacteria**				
*E. coli*	Mesophile	50S (LSU)	2AW4	(50)
		30S (SSU)	2AW7	
*T. thermophilus*	Thermophile	50S (LSU)	3UXR	(51)
		30S (SSU)	3UXS	
*D. radiodurans*	Mesophile	50S (LSU)	2ZJR	(52)
*B. subtilis*	Mesophile	Ribonuclease P	1NBS	(53)
*T. maritima*	Thermophile	Lysine riboswitch	3DIR	(54)
**Eukarya**				
*Tetr. thermophila*	Thermophile	60S (LSU)	4A1B	(55)
*S. cerevisiae*	Mesophile	60S (LSU)	3U5H	(56)
*X. laevis*	Mesophile	5S rRNA	1UN6	(57)
*RAT*	Mesophile	S/R Domain from 28S rRNA	1Q96	(58)

The base pair families and the order in which they occur in S/R motif are conserved, but the sequence varies in different organisms and locations. For example, in almost all eubacterial 23S rRNA the tHH base pair in the H95 motif is AC, while in most Archaeal and Eukaryal LSUs it is AA. In addition, some S/R motifs have one more non-WC base pair forming 6 × 5 loop. Overall, however the H95 motif is very conserved in sequence.

Generally, S/R motifs in other locations display more sequence variability, especially, in the tSH base pair, where we observe seven different base combinations in LSU, SSU and 5S rRNA 3D structures AG, AA, UC, CC, AC, GG and UA (Figures 1 and 2). All these sequence variants, with the exception of G-G, form isosteric tSH base pairs (29).

Unusual substitutions are observed in some S/R motifs. For example, in the *S. cerevisiae* version of the LSU H11 S/R motif, there is an unusual bulged A in place of highly conserved G which forms a cSH/tWH base triple with conserved U and A bases of the motif. Also, an unusual GA tHH base combination is observed in the *H. marismortui* version of 23S rRNA S/R motif that is embedded in the complex junction from domain II.

Within 16S rRNA, S/R-like motifs occur in three places: h27; the three-way junction comprising h28, 29 and 43; and h17 (Figure 2D). These motifs differ from the prototypical structure in that the h27 S/R motif contains a conserved bulged C, the 3WJ S/R motif is missing one tSH base pair and the S/R motif from h17 shares only tHS AG and tWH UA base pairs with the prototype. Nevertheless, the overall geometry of these motifs is similar to the prototypical structure, and we include them in the analysis of sequence variations.

### Base pair isostericity in S/R motifs

Two base pairs are said to be isosteric when they meet three criteria (1): (1) The C1′–C1′ distances are nearly the same; (2) the paired bases are related by identical rotations in 3D space; and (3) H-bonds are formed between equivalent atoms of the bases. Consequently, isosteric base substitutions in RNA 3D motifs are structurally neutral and do not interrupt the 3D geometry, while non-isosteric mutations distort the structure or prevent it from forming. Table 2 represents the observed sequence variations from 3D structures of S/R motif tabulated in 4 × 4 Isostericity matrices. Squares representing isosteric base pairs in the same base pair family are shaded the same color. Different colors indicate different isosteric groups. For example, there are two isosteric groups in the tHS family and almost all sequence variants belong to the same family for both tHS base pairs of the motif. Supplementary Figure S1 compares structural and geometrical parameters of isosteric and near isosteric tHS and tHH base pairs that appear frequently in S/R motifs.

**Table 2.** RNA base pair variations observed in 3D structures of S/R motifs in nonredundant data set^a^

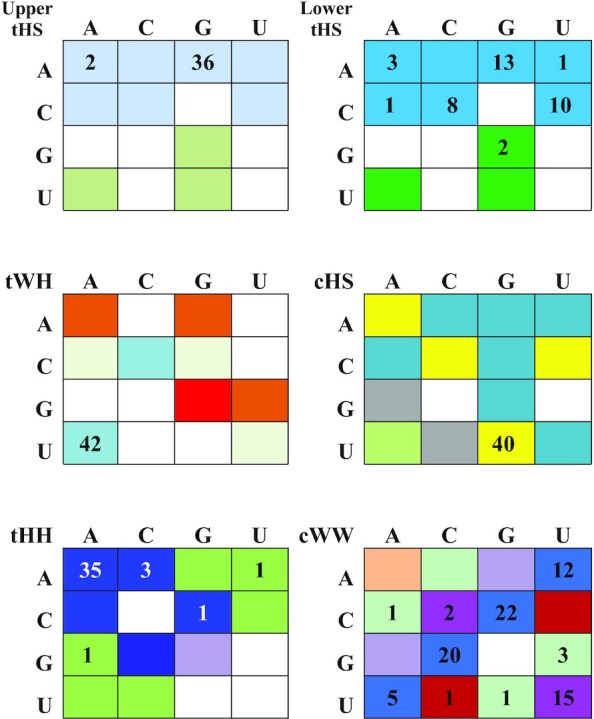

^a^Within each family, the base combinations that from isosteric base pairs are shaded the same color. For example in *t*WH base combinations UA and CC are isosteric and shaded light blue. In addition, each color repressents specific isosteric group as follows:

Blue color indicate i1 group; Green boxes—i2 group; Violet, Orange and Red boxes indicate i3, i4 and i5 groups respectively. Purple boxes indicates i6 group of isosteric pairs. Yellow colored shades indicate combined i1/i2 isosteric pairs within the family. Grey colored cells are modeled interactions, not yet observed in high resolution X-ray (59). Unshaded cell in each table indicate base combinations that do not form that type of base pair.

While substitution of isosteric base pairs does not distort the 3D structure of the motif, it may affect the thermodynamic stability or the rigidity/flexibility of the structure. This aspect of isosteric substitutions in conserved motifs has not been addressed adequately in previous work.

Guided by the structural analysis, we suspected that some of the observed sequence variation in non-WC base pairs in the S/R motifs may be due to thermal adaptation. For example, Figure 2 demonstrates that in both thermophilic organisms (*T. thermophilus*, and *T. maritima*) the upper tHS base pair is always AG, whereas in mesophilic groups AA also occurs (e.g. *E. coli* h27 16s rRNA in Figure 2D and *B. subtilis* RNAse P in Figure 2C). The lower tSH base pair accommodates multiple sequence combinations; however, it is not known to what extent, if any, these variations affect the stability of the S/R motif. For example, there are two N-H^…^N bonds in the tSH GA base pair but only one amine – carbonyl N-H^…^O = C H-bond in the isosteric tSH UC pair. Does this mean that motifs in which GA substitutes for UC at this position are significantly more stable? Are there correlations between the organism's optimal growth temperature and the sequence variations? The present study aims to address these questions.

### Thermodynamic study of RNA duplexes containing sarcin-ricin motifs

**Figure 3.**
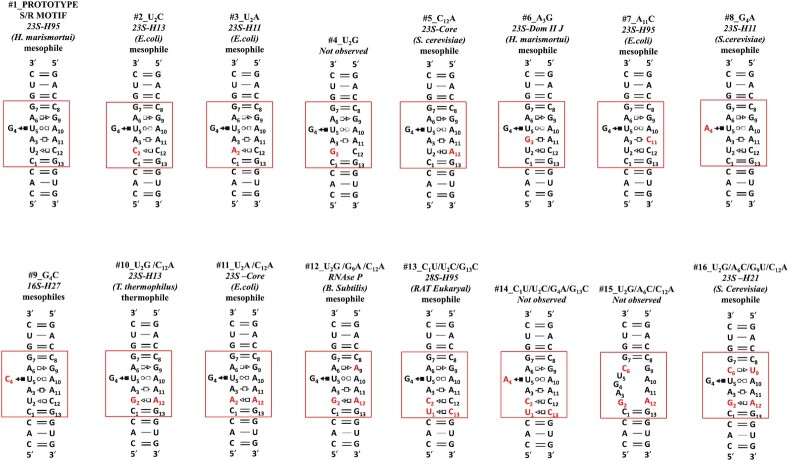
Sequences and schematic structures of the RNA sarcin-ricin motifs used in thermal denaturation studies. Names are given on the top of each duplex as follows: nucleotide mutation in respect to prototype molecule (red colored rectangle), appearance in rRNA with indication of helix number (e.g. *23S-H13*), name of bacteria where sequence appears (e.g. *E. coli)*, and optimal growth temperature organism adapted (e.g. mesophile). The symbols of base pair interactions are given according to N.B. Leontis and E. Westhof nomenclature

Having analyzed sequence variability in the S/R motifs found in the 3D structural database, we designed RNA duplexes (Figure 3) that systematically incorporate the key sequence variations identified by these comparisons. Next, we describe the design of these duplexes and the results of UV-melting experiments that were carried out to quantify their stabilities.

#### Design of sequence variants and nomenclature

For consistency, we numbered each nucleotide in the S/R motif sequences from the 5′ to 3′ direction starting with the left-most strand and including the flanking base pair, as shown in Figure 3 (the red rectangle). Numbered nucleotides are enclosed in red rectangles and nucleotides that differ from prototype in Figure 3 are colored in red in all figures.

Each duplex has its unique number 1 through 16 and is named and labeled to reflect the following:

- Nucleotide substitution(s) relative to the prototype duplex. For example, the '#2_U2C' duplex U at position 2 changed to C. Each nucleotide substitution is colored in red (Figure 3).
- Motif location in rRNA, indicated by the helix number and organisms name, e.g. 23S-H13 *E. coli*.
- Optimal growth temperature class (psychrophilic, mesophilic, thermophilic, hyperthermophilic).

We searched for S/R motif instances in the non-redundant RNA 3D structural database using WebFR3D (26) and identified 14 unique S/R motif sequences (Figure 2). These 4 × 5 internal loops were inserted in RNA duplexes flanked by two canonical base pairs. To prevent sequence end flaying of the flanking helices prior to melting of the motif we terminated each helix with GC Watson–Crick pairs. The sequences were checked with Mfold (www.mfold.bioinfo.rpi.edu, Zuker 2003) to avoid the formation of self-complementary secondary structures, internal hairpin structures or misaligned duplexes. We used duplexes for melting experiments rather than hairpin constructs to avoid specific interactions of hairpins with divalent metal ions (30,31). Moreover, base substitutions are easier and cheaper to design in duplex RNAs and data analysis in duplexes is more robust.

We also added two control molecules that were not observed in 3D structures. These controls contain base substitutions intended to prevent the formation of the S/R motif by disrupting one of the two tHS base pairs. For example, in the #4_U2G molecule U2, which forms a tSH base pair with C12, is substituted by G. The resulting GC juxtaposition is not expected to form a tSH pair since GC is never observed to form this type of base pair in any 3D structure. On the other hand, the GC combination can form a canonical and stable Watson–Crick base pair, which is expected to disrupt the formation of the S/R motif. Likewise, the A6C substitution in the #16_U2G/A6C/C12A is expected to disrupt the formation of the other tHS base pair and thus the whole motif by producing a CG between positions 6 and 9 of the motif.

All 16 designed RNA duplexes, fourteen of which are observed in 3D structures and two of which are unobserved controls are shown in Figure 3 with differences from the prototype indicated by red rectangles.

### Selecting S/R motif flanking base pairs

The flanking Watson–Crick base pairs of S/R motifs can vary. In addition to the canonical cWW GC and AU flanking pairs, in 3D structures cWW GU, CC, UC, UU and CA base pairs also occur (Figure 2 and summarized in Table 2). It is not possible to perform thermodynamic studies on all variations of flanking pairs. Thus, we designed a controlled study varying one flanking cWW pair of the motif (molecule #10_ U2G/C12A). We kept the G7C8 closing base pair constant and tested four different variants of the other closing base pair (C1G13, G1C13, U1G13 and U1A13). The results of these UV-melting studies are shown in Table 3. Except for the wobble U1G13 flanking pair all variants showed distinct transition curves. For duplex design we chose the C1G13 cWW base pair for two reasons. First, this variant has a high melting temperature of 37.3°C, and secondly, it is easier to purify this molecule on TLC plates because it has fewer guanines on the left strand.

**Table 3.** Effect of flanking base pairs on stability of S/R motif

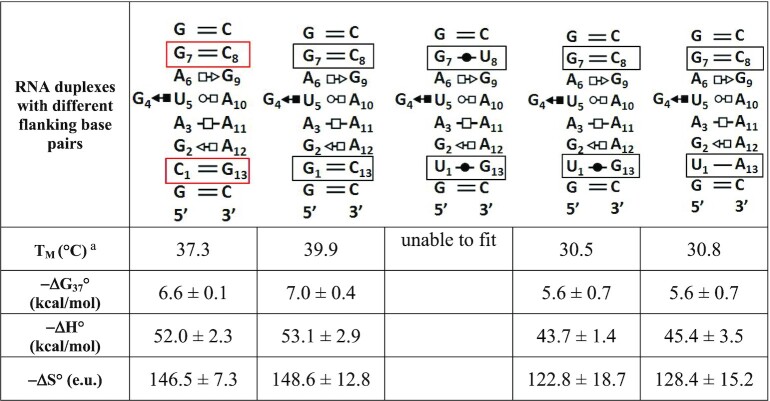

^a^RNA duplexes were melted in 10 mM Sodium Cacodylate buffer pH = 6.9, 0.5 mM EDTA, 100 mM NaCl, 10mM MgCl_2_. Tm values calculated at 100 μM total RNA concentration.

### UV-melting of duplexes containing S/R motifs

UV-melting experiments of S/R motif RNA duplexes were performed by monitoring of the absorbance change at 260 nm. Some RNA melting curves were also recorded at 280 nm as a check and gave similar results. Melting controls of single strands did not exhibit distinct transitions. Table 4 shows the thermodynamic parameters for duplex formation that were obtained from averaging the independently fitted melting curves to the two-state model, adjusted to 100 μM Ct with the assumption that extinction coefficients for single strands depend linearly on temperature. To confirm two state behavior we generated a Van’t Hoff plot of *T*_M_^−1^ versus log (Ct/4) of the #10_U2G/C12A duplex and obtained agreement with calculated parameters within 10% for the two methods, as shown in Supplementary Figure S2, validating the two state assumption.

**Table 4.** Thermodynamic parameters for S/R motif duplex formation at 10 mM MgCl_2_,100 mM NaCl**^;^** and at 1 M NaCl

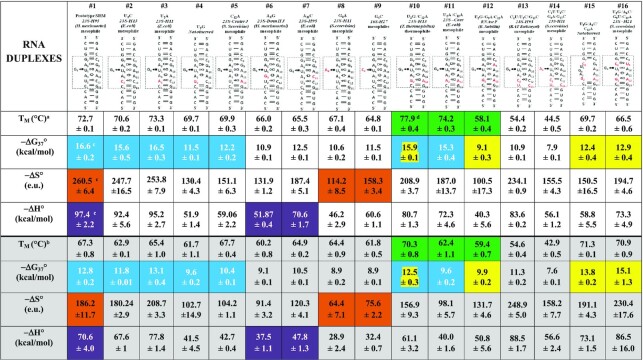

^a^RNA duplexes melted in 10 mM Na^+^ Cacodylate buffer pH 6.9 in presence of 10 mM MgCl_2_ and 100 mM NaCl, *T*_M_ values calculated at 10^−4^ M oligomer concentration (Ct) using average curve fits of at least 4 replicate experiments.

^b^RNA duplexes melted in 10 mM Na^+^ Cacodylate buffer pH 6.9 in presence of 1 M NaCl, *T*_M_ values calculated at 10^−4^ M oligomer concentration (Ct) using average curve fits of at least four replicate experiments.

^c^Duplexes which are different only in upper *t*HS pairs are in yellow shaded cells; lower *t*SH pairs are in blue colored cells; ‘bulges’ nt in brown colored cells; *t*HH pairs are in violet colored cells.

^d^The green shaded cells correspond to differences of upper and lower *t*HS A–G base pair combination.

#### Mg^2+^ and Na^+^ buffers

It is standard to perform UV melting experiments of RNA duplexes in the presence of 1 M NaCl, to compensate for the absence of divalent ions. However, 3D motifs like S/R do not fold correctly in the absence of divalent ions. Defined conformation of RNA bases prior to melting is crucial and often depends on the addition of divalent ions. Serra et. al. showed that the stability of RNA structural motifs depends on Mg^2+^ ions, in particular, a melting temperature increase in the presence of 50 mM MgCl_2_ has been demonstrated for eukaryotic 5S loop E (32). Moreover, Brownian dynamic simulation studies suggested three potential but delocalized metal-binding sites in the S/R motif (33). This was confirmed by extensive molecular dynamic simulation (34). Therefore, to investigate the role of Mg^2+^ on the S/R motif RNA duplex stability we performed UV-melting studies in two distinct buffer compositions, one with Mg^2+^ and one without:

1. 10mM Sodium Cacodylate buffer pH 6.9, 0.5 mM EDTA with 0.1 M NaCl and 10 mM MgCl_2_.
2. 10mM Sodium Cacodylate buffer pH 6.9a, 0.5 mM EDTA and 1 M NaCl.

The differences in thermodynamic parameters of RNA duplexes from 10 mM Mg^2+^ and in 1M NaCl are summarized in Table 3 (grey shaded cells correspond to experiment with no Mg^2+^ ions). The data suggest that majority of RNA duplexes generally have higher stability in Mg^2+^ containing buffer. The differences in thermodynamic parameters obtained between two buffers are summarized in Table 4.

**Figure 4.**
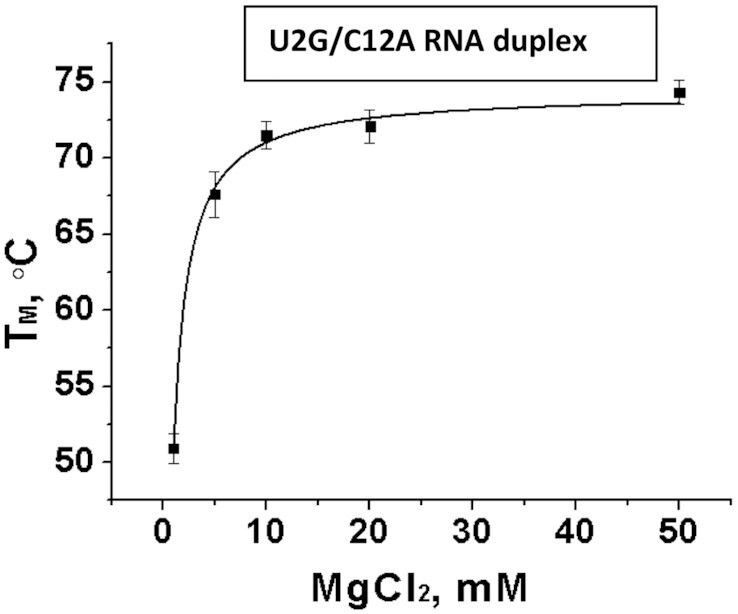
Melting temperature of reference duplex as a function of [Mg^2+^]. At 10 mM MgCl_2_, the *T*_M_ curve reaches its plateau.

**Figure 5.**
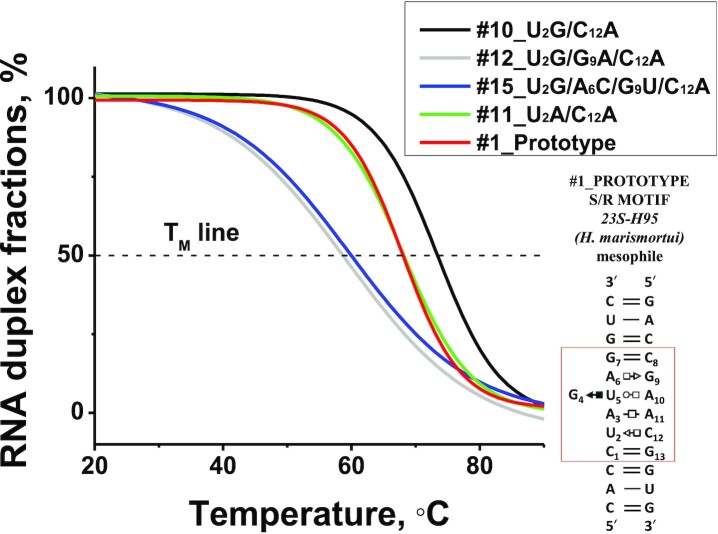
Representative absorbance versus temperature profiles of five RNA duplexes melted in sodium cacodylate buffer pH 6.94, 10 mM MgCl_2_ and 0.1 M NaCl. Corresponding names are given in legend. 2D structures of these molecules could be found in Figure 3.

To find the optimal Mg^2+^ concentration for the S/R motif formation we measured *T*_M_ of the #10_U2G/C12A RNA duplex as a function of [Mg^2+^] from 1 to 50 mM. Figure 4 shows that at 10 mM MgCl_2_ the *T*_M_ curve reaches its plateau and further increase in [Mg^2+^] does not significantly affect the *T*_M_. Thus, we used 10 mM MgCl_2_ in all experiments involving magnesium. Typical thermal denaturation curves of five representative RNA duplexes containing S/R motif sequence taken at 10 mM MgCl_2_ are shown in Figure 5. The samples containing Mg^2+^ were only melted once due to Mg^2+^ driven RNA cleavage.

### Influence of tHS base pair combinations on S/R motif duplex stability

The tHS base pair family frequently occurs in functional RNA motifs including S/R motif, Kink-turn, loop E, multi-way junctions as well as hairpin loops (e.g. GNRA hairpin loop). The most well known member of this family is the tHS A–G pair, also known as sheared AG base pair which often contributes to RNA stability by extensive cross-strand purine stacking.

Sarcin/Ricin motifs contain two conserved tHS base pairs with opposite orientations. We refer to the tHS pair between nts 6 and 9 as the ‘upper tHS’ pair and the pair between 2 and 12 as the ‘lower tSH’ pair.

#### Upper tHS position

Four variants of the upper tHS pairing position (nt 6 and 9) were studied:

A6–G9, A6–A9, C6–U9 and C6–G9 corresponding to molecules #10, #12, #15 and #16 respectively. In all these duplexes the lower tSH position was G2–A12 (see Table 4, yellow shaded cells). Three of these combinations are isosteric with the prototype and should preserve the conformation of S/R motif. The fourth variant juxtaposes C6 and G9, which cannot form the tHS pair and likely forms cWW, which is expected to disrupt the motif structure. Comparison of their thermodynamic parameters reveals the following results: the most stable RNA duplex is #10_U2G/C12A, which has A6-G9 with Δ*G*°_37_ of –15.9 kcal/mol in the presence of Mg^2+^ ions. This tHS base combination is the most common in S/R motifs according to WebFR3D results (Summarized in Table 2). The single mutation in the upper tHS position in molecule G9A generates the isosteric A6–A9 tHS base pair. This substitution dramatically decreased the stability of the RNA duplex by ∼6 kcal/mol compared to tHS A6–G9 (see entry for the #10_U2G/G9A/C12A duplex in Table 2). The intrinsic energies for these isosteric tHS AG and tHS AA pairs were compared previously (35) using quantum chemical calculations and it was found that the tHS AG base pair is substantially more stable than tHS AA consistent with our observations. Molecule #15_U2G/A6C/G9U/C12A with isosteric C6–U9 has a Δ*G*°_37_ value of –12.9 kcal/mol which is also less stable than A6–G9, but more stable than A6–A9.

The stability of the control #16_U2G/A6C/C12A S/R motif variant which juxtaposes C6–G9 pair has a Δ*G*°_37_ value of –12.4 kcal/mol, which is similar to the #15_U2G/A6C/G9U/C12A duplex containing tHS C6–U9. However, the melting experiment performed in 1 M NaCl resulted in lower Δ*G*°_37_ of –13.8 kcal/mol for this juxtaposed C6-G9 control pair (Table 4, lower panel yellow shaded cells).

Interestingly, presence of 1 M NaCl also stabilizes the formation of RNA duplexes containing tHS C6-U9 and A6–A9 by 2.2 kcal/mol and 0.8 kcal/mol respectively (Table 5). The presence of Mg^2+^ ions favors only the tHS A6–G9 pair by ΔΔ*G*° = –3.4 kcal/mol. To summarize, the isosteric upper *t*HS base pair combinations can be ordered by stability as follows:

upper *t*HS with Mg^2+^ : A6–G9 > C6–U9 > A6–A9
upper *t*HS no Mg^2+^: C6–U9 > A6–G9 > A6–A9

**Table 5.** Effect of Mg^2+^ ions in stabilization of S/R motif duplex^a^

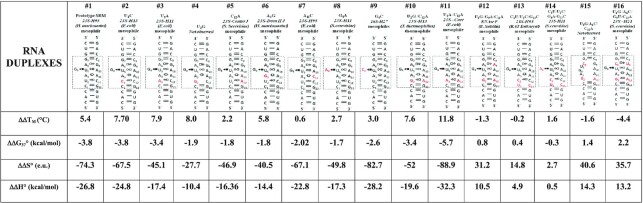

^a^Values obtained by subtracting S/R motif data (see Table 5) melted in presence of MgCl_2_ and its absence. For example for prototype S/R motif molecule: ΔΔ*G*_37_° = Δ*G*_37_°_Mg2+_ – Δ*G*_37_°_1 M NaCl._

#### Lower tSH position

The lower tSH base pair position exhibits much more sequence variability than the upper tHS (see Table 2). We studied seven RNA duplexes (#1, #2, #3, #4, #5, #10 and #11) that are distinct in only lower tSH base pair combinations. They are tSH U2–C12 (prototype), C2–C12, C2–A12, C2–G12, A2–U12, A2–G12 and A2–A12. The calculated Δ*G*°_37_ parameters for these duplexes are shown in the Table 4 and are indicated as blue colored cells for clarity.

Data shows that many of these duplexes have relatively similar Δ*G*°_37_ of ∼15.5 kcal/mol and the order of stability follows:

tSH U2–C12 (Δ*G*°_37_ = –16.6 kcal/mol) > tSH A2–C12 (Δ*G*°_37_ = –16.5 kcal/mol) > tSH G2–A12 (Δ*G*°_37_ = –15.9 kcal/mol) > tSH C2–C12 (Δ*G*°_37_ = –15.6 kcal/mol) > tSH A2–A12 (Δ*G*°_37_ = –15.3 kcal/mol) >> tSH U2–A12 (Δ*G*°_37_ = –12.2 kcal/mol).

All lower tSH pairs are isosteric (see above). And only tSH U2–A12 is ambiguous. The A at position C12 could form a Watson–Crick base pair with U2, which is expected to disrupt the motif. However, UA is also an allowed base combination for tSH, and so it is possible that the tSH pairing geometry is conserved. This sequence variant tests the resilience of the motif to ambiguous base substitutions. In fact, this sequence variant occurs in the central junction of yeast 26S rRNA as shown in Figure 2. In the 3D structure (PDB ID: 3U5H) we observe the U and A forming the tSH geometry, so we expect this variant to form the motif.

On the other hand, the control molecule #4_ U2G comprises tSH G2–C12. The tSH GC combination has never been observed in any 3D structure and thus is not expected to form this combination. UV-melting data shows that this RNA duplex has a Δ*G*°_37_ of –11.5 kcal/mol and suggests that this substitution significantly reduces the stability of the S/R motif.

Comparison of the lower tSH isosteric sequence combinations of the S/R motif RNA duplexes melted in the presence of 1 M NaCl shows a slightly different order of stability as compared to the experiment with 10 mM Mg^2+^:

tSH A2–C12 (Δ*G*°_37_ = –13.1 kcal/mol) > tSH U2–C12 (Δ*G*°_37_ = –12.8 kcal/mol) > tSH G2–A12 (Δ*G*°_37_ = –12.5 kcal/mol) > tSH C2–C12 (Δ*G*°_37_ = –11.8 kcal/mol) > tSH U2–A12 (Δ*G*°_37_ = –10.4 kcal/mol) > tSH A2–A12 (Δ*G*°_37_ = –9.6 kcal/mol).

Interestingly, all isosteric combinations at the lower tSH depend on the presence of Mg^2+^, and, generally Mg^2+^ ions stabilize the S/R motif of these duplexes, while we do not observe this effect at the upper tHS position.’ ‘Moreover, while isosteric base pair substitutions at the lower tSH position do not significantly impact the stability of S/R motif, substitution in the upper tHS may depending upon the sequence. For example, comparison the free energy parameters between RNA duplex #10_U2G/C12A with upper and lower tHS A–G and #11_U2A/C12A RNA duplex with upper tHS A6-G9 but lower tSH A2–A12 shows the difference in ΔΔ*G*° is small, only –0.6 kcal/mol (15.9–15.3). However, comparison between molecules #10_U2G/C12A and #12_U2G/G9A/C12A with upper tHS A6–A9 and lower tSH G2–A12 shows a much larger difference in ΔΔ*G*° of –6.8 kcal/mol (see Table 4, their *T*_M_ are in green colored cells)

**Figure 6.**
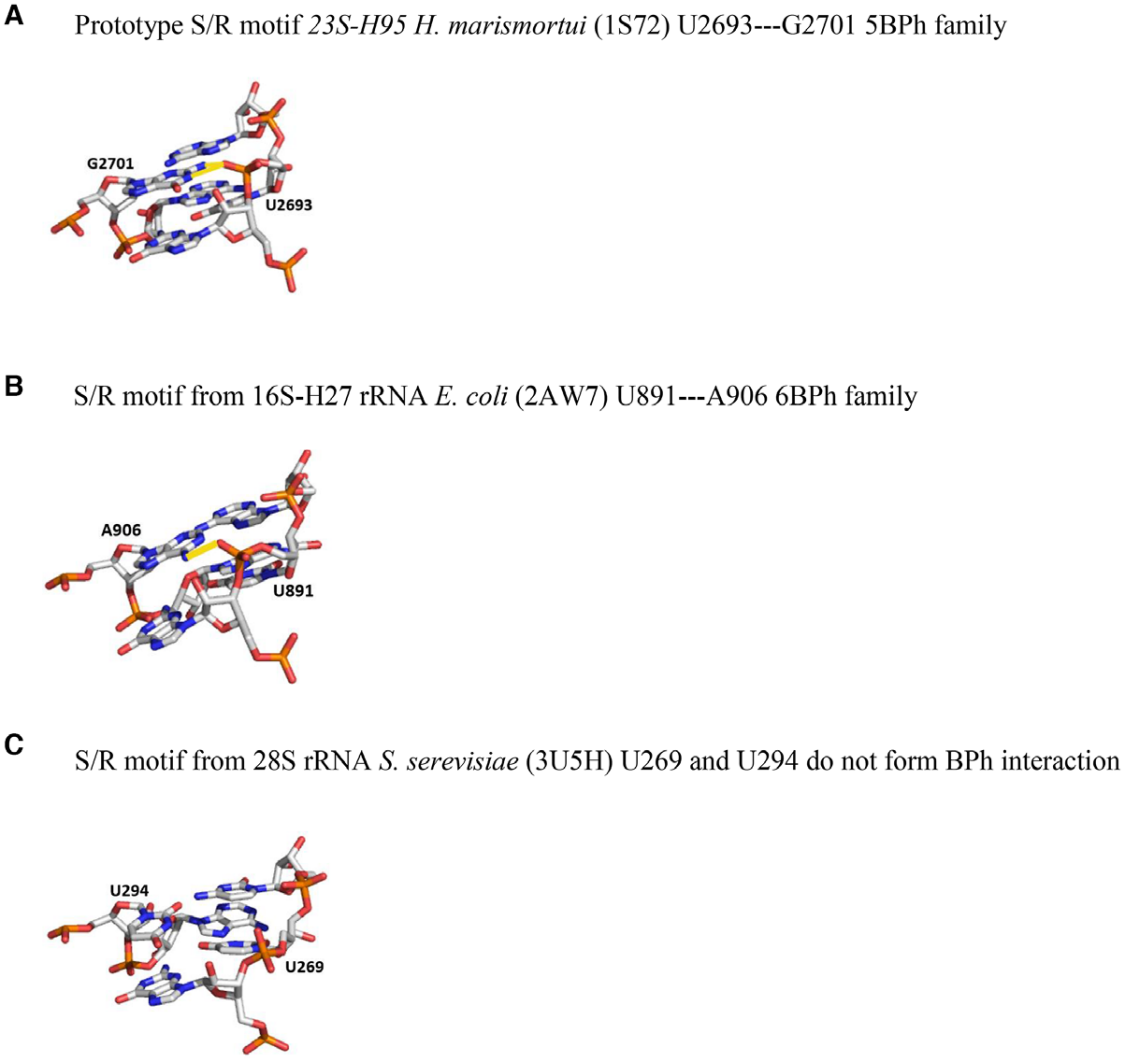
Examples of BPh interactions between upper tHS and triplet U. When the upper tHS is AG there is a hydrogen bonding between oxygen of phosphate group of U and aminogroup of G which belongs to 5BPhfamily (**A**). When the upper tHS is AA then interaction between the phosphate group of U and aminogroup of A belongs to 6BPh family (**B**). There is no BPh interaction when the upper tHS has CU base pair combination (**C**).

So why do we observe such big changes in stabilities of RNA duplexes mutated in the upper tHS and not in the lower tSH? The stability of S/R motif must be defined by the context of tHS base pair family within the structure. ‘For example, while lower tSH base pair stacked between tHH and cWW pairs, the upper tHS stacked between cWW on one side and tWH pair of the triple on the other. These different nearest neighbor nucleotides of the upper and lower tHS pair, in fact, could explain the overall duplex stability.’ In addition, the stability of S/R motif structure depends not only on the stacking interactions but also on base-phosphate (BPh) interactions (36,37). 3D structures of S/R motifs with different upper tHS base pair combinations revealed formation of different BPh hydrogen bonding as demonstrated in Figure 6. This figure shows that in the prototype S/R motif, nucleotide U2693, a part of the triplet, interacts with nucleotide G2701 forming 5BPh interaction. The same hydrogen bond between corresponding nucleotides U891 and A906 in S/R motif containing upper tHS AA belongs to 6BPh family. This suggests that BPh interactions might be crucial in stabilizing the S/R motif. In fact, the importance of BPh interactions was pointed out by Zirbel *et al.* (37), and revealed their evolutionary roles in ribosomal RNA. It was shown that ∼12% of nucleotides in large RNAs are involved in direct BPh interactions, and these interactions are the most conserved during evolution. This may explain why we see such large differences in stability when the upper tHS varies. Surprisingly, while there is no BPh interaction between nucleotides U269 and U294 when the upper tHS is C–U, as shown in Figure 6, these duplexes are more stable than tHS A–A.

### Bulged nucleotides stabilize the S/R motif duplex by high entropy contribution

**Figure 7.**
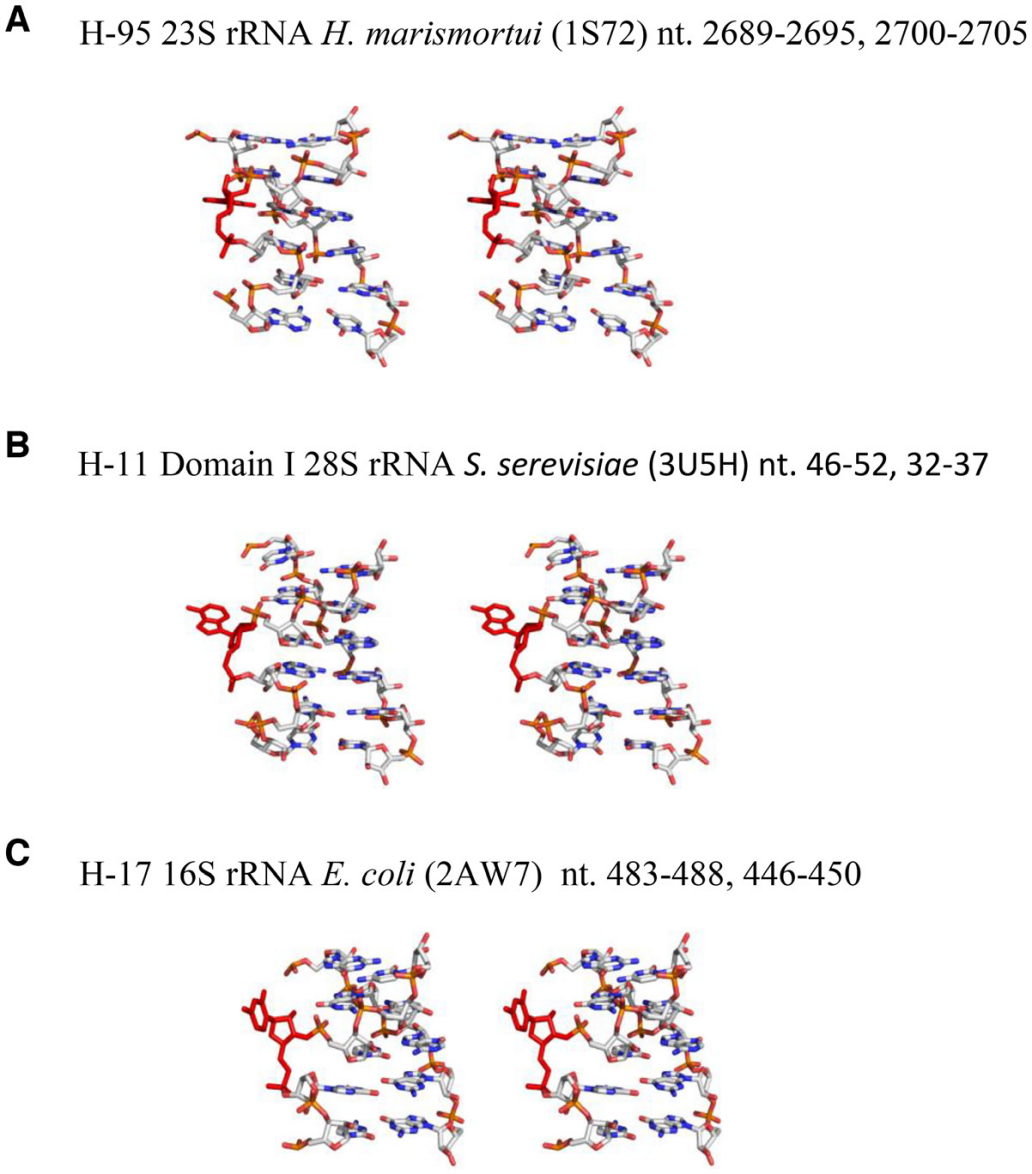
Comparison of structures of S/R motifs varying the bulged base. In most S/R motifs the bulged base is G, as exemplified in the S/R motif from H-95 of 23S rRNA (Panel **A**). The G is always observed to form a base triplet and base phosphate interaction. When this G is replaced by A (Panel **B**) or U panel (**C**), the base is no longer observed to form these interactions.

We studied three S/R duplexes (#1, #8 and # 9) that have differences only in the bulged nt G4, A4 and C4 (Table 4 in brown shaded cells). The prototype RNA duplex with highly conserved bulge G4 revealed the lowest Δ*G*°_37_ value of –16.6 kcal/mol, while Δ*G*°_37_ for the S/R motifs with bulges A4 and C4 were found to be –10.6 kcal/mol and –11.5 kcal/mol respectively. The free energy difference of the bulged G4 compared to A4 and C4 is ∼–5.5 kcal suggesting that the prototype duplex much more stable than A4 and C4 (duplexes G4A and G4C). However, mutations in bulged nucleotides result in significant differences in calculated entropy values. The calculated entropy values for these duplexes are shown in Table 4 in brown colored cells for clarity. The prototype motif with the bulged G4 has a Δ*S*° of –260.5 e.u. while substitution to bulged A4 or C4 resulted in significantly higher Δ*S*° values of –114.2 e.u. and –158.3 e.u. Even larger entropy change was observed in presence of 1 M NaCl for A4 and C4 (see Table 4). Evidently, the high entropy values for S/R motifs containing bulged a A4 or C4 contributes to the overall stability of the entire duplex. Why there is a big difference in entropy parameters of A4 and C4 compare to prototype G4? The survey of 3D structure demonstrates that A and C bulged nucleotides in the S/R motif context are very rare (Figure 2). As shown in the crystal structure of 23S rRNA in H-11 of *S. cerevisiae*, the bulged A does not participate in the triple interaction and the nucleotide is bulged out from the motif entirely suggesting the flexibility of entire S/R motif. The same pattern is observed for the bulged U (not used in our studies) at equivalent position from S/R motif of 16S rRNA of H17 (Figure 7). Moreover, the previously reported NMR data also suggest that the replacement of bulged G to A destabilizes the internal loop while overall conformation appears to be similar (38). Our data is consistent with reported observations in the literature and demonstrates that even if the bulged A or C do not participate in triplet interaction, they help to stabilize the formation of S/R motif by a large entropy contribution.

### Contribution of *trans* Hoogsteen/Hoogsteen base pair combinations to stability of S/R motif

Three RNA duplexes (#1, #6 and #7) were studied in which only tHH the base combination differed: tHH A3-A11, tHH G3-A11 and tHH A3-C11 (Table 4, violet shaded cells). The calculated Δ*G*°_37_ revealed that the tHH A3-A11 base pair combination is more stable than tHH A3–C11 and the least stable is tHH G3-A11. The order of stability is well correlated with the occurrences of tHH base combinations, as tHH AA is the most frequent in existing 3D structures (Figure 2, and summarized in Table 3) and most stable. These data clearly demonstrate that the tHH base pair is important in the S/R motif, as a single mutation of the tHH A3-A11 pair combination to tHH A3–C11 or tHH G3–A11 influences the overall motif stability. In addition, the stability of the S/R motif with tHH G3–A11pair is entropically driven as its Δ*S*° value of –131.9 e.u. is significantly higher than that of tHH A3–A11, Δ*S*° = –260.5, and tHH A3–C11, Δ*S*° = –187.4 in presence of MgCl_2_.

### Structural probing of S/R motifs

Next, to determine which of the sequence variants studied by thermodynamic methods likely form the S/R motif, we employed established enzymatic and chemical probes, including RNases T1, A and V1 and the chemical probe DEPC (diethylpyrocarbonate).

**Figure 8.**
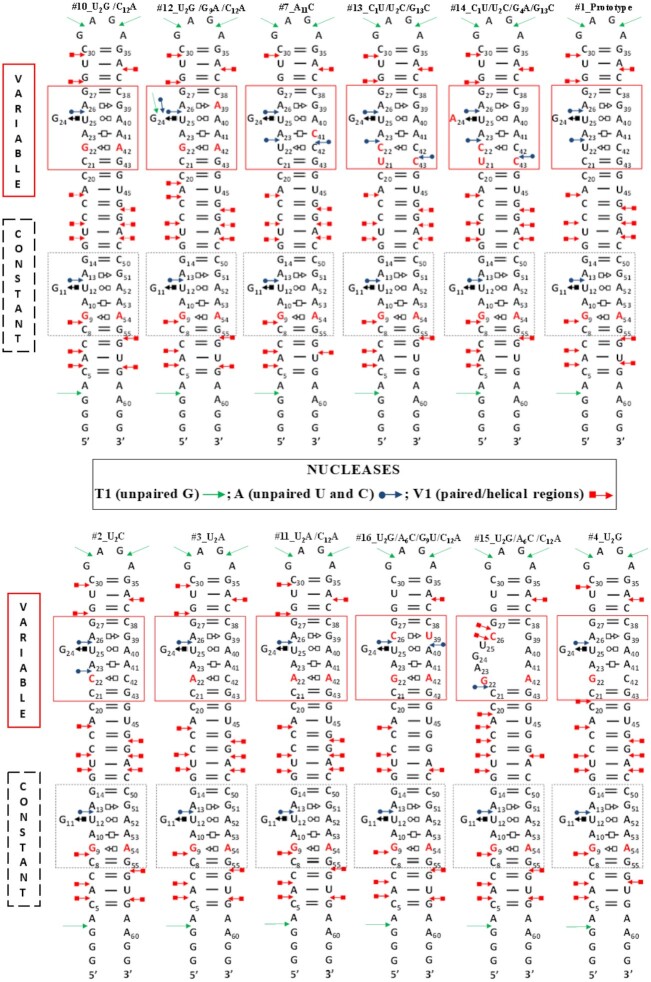
2D diagrams of RNA hairpins used in structure probing experiments. These molecules consist of the same sequences of GAGA hairpin loop and have two distinct sequences regions: (i) constant region (highlighted as a puncture black rectangle) corresponds to sequence of molecule #10_U2G/C12A which conserved among all molecule and (ii) variable regions (highlighted as a red rectangle) implemented as a signature of individual molecule. The variable region contains sequences of duplexes used in thermal denaturation experiment. Probe RNA molecules are named according to the names of short duplexes inserted in variable region. For instance, prototype S/R motif has this name because the sequence of prototype S/R motif short duplex was implemented to the variable region. The summary of the nuclease digestion experiments is also shown. Cleavage patterns of RNA hairpin secondary structures is represented by symbols corresponding to the legend on center.

To directly probe the RNA duplexes used in UV-melting studies is not feasible, so we designed longer hairpin shaped molecule which included a control motif for probing experiments. The sequences of twelve of the sixteen RNA constructs designed for structure probing are shown Figure 8. Each contains a stable GAGA hairpin tetra-loop, which was intended to form a standard GNRA structure. All constructs are 62 nucleotides long. Using the hairpin design we were able to implement an efficient method for S/R motif structure probing: Each probing construct has a constant (control) and a variable region, corresponding to S/R motif sequences from the melting experiment as shown in Figure 8. The S/R motif closest to the 5′ and 3′ ends of each construct is the control motif (enclosed in the dotted-line rectangle in Figure 8) and corresponds to the sequence of the #10_U2G/C12A S/R variant. It is the same in all molecules and serves as a control for establishing the relative intensities of the cleavage when probing different molecules under different conditions. The motif closest to the hairpin loop is variable and is highlighted in a red rectangle in Figure 8. It corresponds to the different sequence variants inserted in RNA duplexes for UV-melting studies. This design strategy allows us to analyze the probing results for all RNAs simultaneously as the same control S/R motif is presented in each construct.

### Structure probing by nucleases

**Figure 9.**
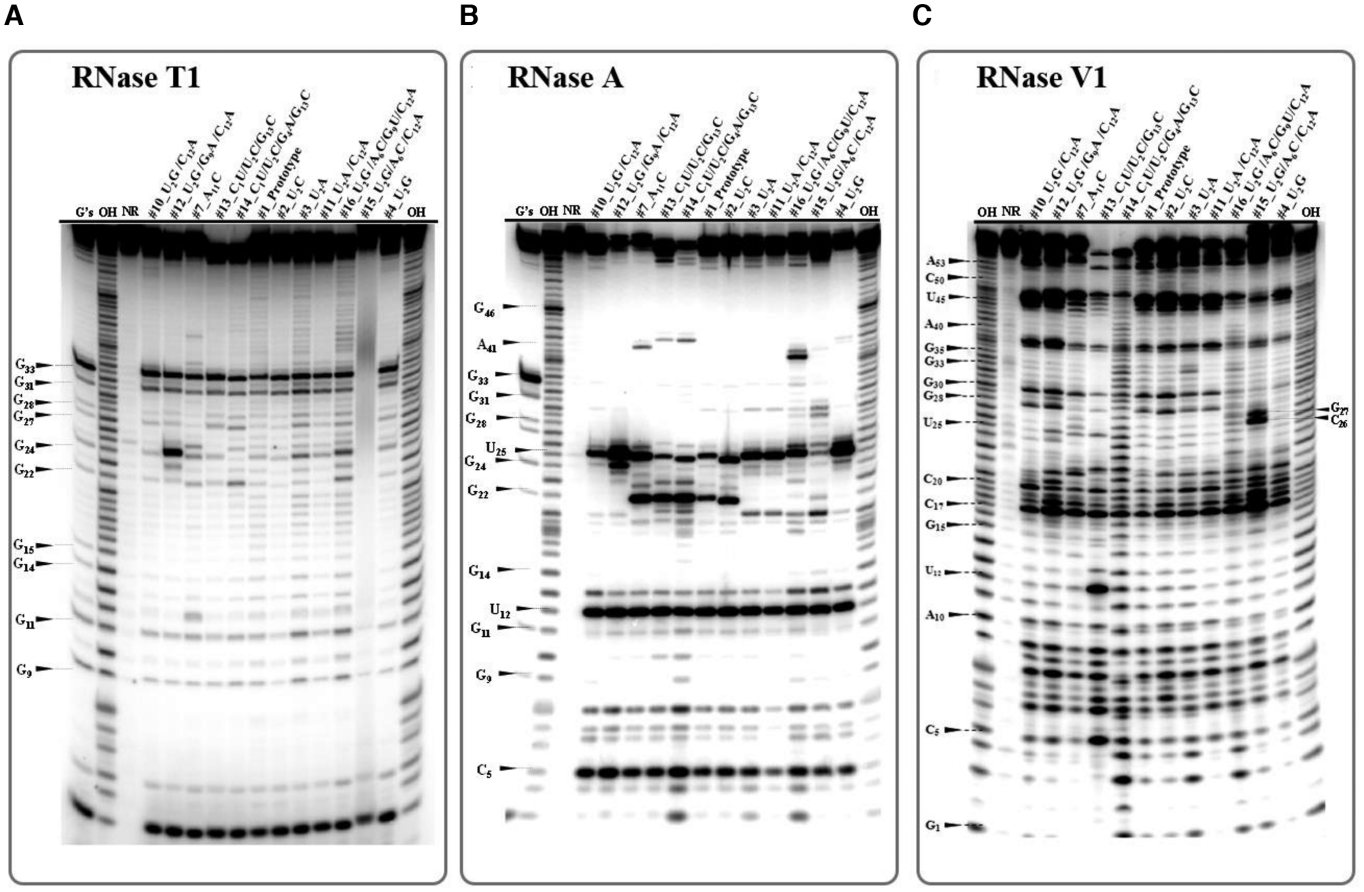
Representative autoradiograms of 15% denaturing gel and cleavage pattern of RNA hairpins by nucleases. The data represents cleavage pattern of 5′-end labeled RNA hairpins treated with specific nucleases shown on the top of each panel: (**A**) RNase T1, (**B**) Rnase A, and (**C**) RNase V1. Lanes: ‘G’s’ is RNase T1 sequencing reaction in presence of 8M urea; ‘OH’ are - base hydrolysis ladders; ‘NR’ is nuclease free RNA molecules (control); other lanes correspond to the names of individual RNA molecules treated with nucleases.

RNA structure probing was carried out with the helix specific RNase V1 and two single-strand specific nucleases T1 and A. RNAs were 5′-end labeled with ^32^P phosphate. All RNAs were probed in their native conformations in the presence of 10 mM MgCl_2_. The buffer conditions and annealing procedures were identical to the UV-melting experiments described above to ensure that the RNA molecules adopt the same conformation. The cleavage data averaged over several experiments are summarized in Figure 8 and corresponding autoradiogram images are provided in Figure 9.

#### RNase T1

The cleavage pattern of RNA hairpins treated with RNase T1 is indicated by green arrow in Figure 8 (for cleavage pattern see Figure 9A). These data show that in all constructs, nucleotides G33 and G31 in the hairpin loop are the most sensitive to RNase T1 digestion and likely are most accessible to enzymes. These nucleotides are located in the hairpin loop region and the GAGA sequence could fold into GNRA-type structure. The difference in sensitivity between G33 and G31 to RNase T1 suggests that G33 remains unpaired, while G31 makes some interaction, consistent with 3D structures of known GNRA motifs, which shows that G33 is unpaired while G31 forms a *t*SH non-WC base pair with A34 (39,40). Thus, the probing data is consistent formation of the GNRA motif as intended in our design.

All other G’s in the probed constructs remained unreactive to RNase T1 except for G24 in the molecule that contains the #12_U2G/G9A/C12A sequence variant. Nucleotide G24 corresponds to the bulged G that forms a cSH pair with U25. In this hairpin, the base pair that stacks on the cSH G24–U25 is tHS A26–A39 instead of tHS A26–G39. The increased sensitivity observed for G24 suggests that tHS A26–A39 pairing makes the S/R motif more flexible and thus lowers its thermostability. This is consistent with the data obtained from UV-melting analysis for the duplex containing the tHS A6–A9 and tHS A6–G9 pairs, demonstrating that the A6–G9 pair favors formation of the S/R motif by Δ*G*°_37_ ∼ -6 kcal/mol compared to the tHS A6–A9 pair (see Table 4).

#### RNase A

To further characterize the flexibility of S/R motifs we employed RNase A which is specific for unpaired pyrimidine nucleotides. The RNase structure probing data are summarized in Figure 8, using blue arrows to indicate the cleavage sites, and Figure 9B. The cleavage pattern obtained after subsequent treatment of hairpins with the RNase revealed consistent cleavage of U12 across all S/R motifs, suggesting structural similarity in the constant region of all constructs. U12 participates in the base triple and is very conserved among all S/R variants obtained from crystal structures (Figure 2). However, we observed different cleavage patterns in the variable regions of hairpins. The conserved U25 in the variable region was sensitive to RNase A among all hairpins with the exception of the hairpin #16_U2G/A6C/C12A (lane #16_U2G/A6C/C12A in figure 9B). As discussed above, this hairpin sequence was designed to disrupt the S/R formation thus we assume that the U 25 forms a canonical cWW pair with A40, which prevents the cleavage of U25. This indicates that mutation of tHS A26–G39 to the non-isosteric C26-G39 pair distorts the S/R motif conformation and thus the stability is reduced (Table 4).

U’s are observed to be sensitive to RNase A when they are present in tHS basepairs (Figure 8). Interestingly, the C’s in the tSH C22-C42 and U22-C42 pairs are not sensitive to RNase A, except when there are two adjacent Y–Y tSH pairs (molecules #13_C1U/U2C/G13C and #14_C1U/U2C/G4AC/G13C). The RNase A data provides evidence that the G22–C42 juxtaposition does not affect the formation of the base triple, because U25 shows the same strong cleavage as in the other sequence variants. These results are consistent with those obtained by RNase A treatment of 5S rRNA from *X. laevis*, where loop E also forms an S/R motif (41). The U that participates in the base triple UAG interaction was found to be sensitive to RNase and is very conserved in S/R motifs (42).

#### RNase V1

Strong RNase V1 cuts are observed in all regions of the RNA constructs that we expected to form Watson–Crick helices (Figure 8 red arrows and Figure 9C). The regions of constructs that contain the S/R and hairpin motifs are resistant to nuclease V1 cuts. Only in the #16_U2G/A6C/C12A construct are the nucleotides U25 and C26 susceptible to cleavage. This is additional evidence that the hairpin containing disrupting mutations has a distinctly different structure in variable sequence region.

Overall, these results are consistent with all 12 designed molecules folding into the intended long hairpin conformation with two internal loops and with U25 forming a non-Watson–Crick base pair in all cases except for the disrupting molecule where it is likely forming a Watson–Crick conformation instead.

### Chemical probing

**Figure 10.**
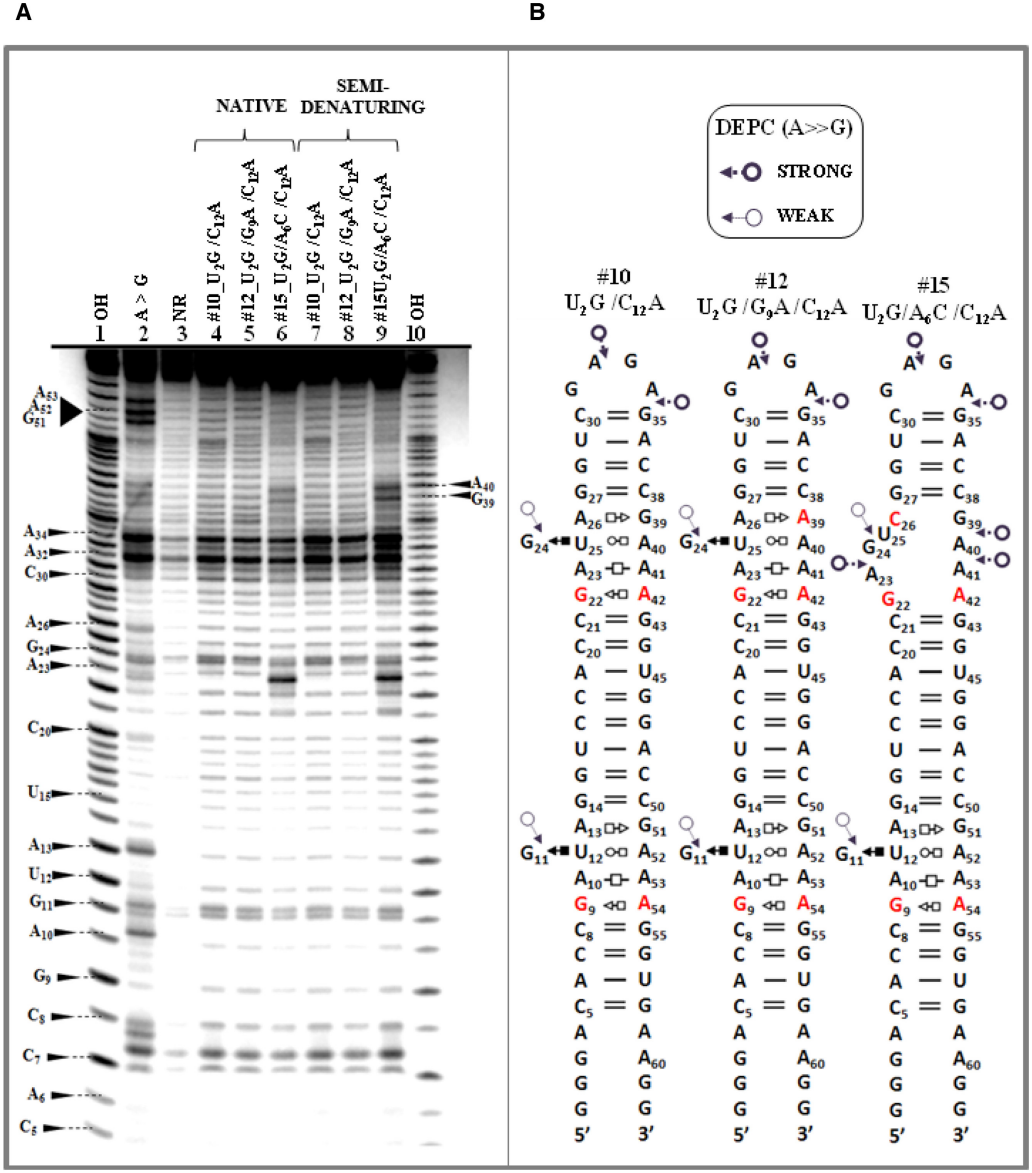
Autoradiogram and summary of chemical probing analysis. (**A**) Structure probing using DEPC to probe accessibility of N-7 positions of Hoogsteen edges of A and G residues. Lanes: 1 and 10 are ‘OH^−^’: alkali hydrolysis step ladder of U2G/C12A hairpin, 2 is 'A > G'- Maxam-Gilbert sequencing reaction, 3 is non-reacted (‘NR’) U2G/C12A RNA, treated with aniline as control; NATIVE 4,5 and 6 are RNAs U2G/C12A, U2G/G9A/C12A, and U2G/A6C/C12A - samples treated with DEPC under native conditions (80mM NaCB buffer pH 6.94, 100mM KCl, 10 mM MgCl_2_, 0.5 mM EDTA); SEMI-DENATURING 7, 8 and 9 are RNAs U2G/C12A, U2G/G9A/C12A and U2G/A6C/C12A -samples treated with DEPC under semi-denaturing condition (80mM NaCB buffer pH 6.94, 100 mM KCl, 0.5 mM EDTA). (**B**) Summary of DEPC sensitivity given by legend on the top of the 2D structures.

The reactivity of the N7 purine positions of three sequence variants #10_U2G/C12A, #12_U2G/G9A/C12A and #16_U2G/A6C/C12A was probed with DEPC. This probe reacts more strongly with A/N7 than G/N7. The reactivity of the adenine residues was determined by subsequent aniline cleavage of 5′-end labeled RNA hairpins. An autoradiogram image and the results from several such experiments are provided in Figure 10.

Comparison of chemical reactivity data for variable and constant regions of the hairpin constructs indicates that the conformation depends on sequence. DEPC accessibility of A32/N7 and A34/N7 in the hairpin loop is very similar for all studied RNAs. This is also true for Gs at position 11 and 24 located in the core base triple. Moreover, we do not observe a significant difference in cleavage patterns of RNAs between native and semi-denaturing conditions. Under the semi-denaturing condition, where the Mg^2+^ ions were eliminated from reaction buffer, only slightly increased cuts were observed.

The variable region in the variant #16_U2G/A6C/C12A is designed to disrupt the S/R motif by preventing the upper tHS base pair from forming, because C26-G39 juxtapositions do not form tHS pairs. As expected, the #16_U2G/A6C/C12A hairpin behaves very differently in the variable region (Figure 10B, lanes 6 and 9). In addition to G24 reactivity, nucleotides G39 and A40 become fully reactive with DEPC in native and semi-denaturing buffers indicating that this internal loop does not have a stable conformation (Figure 10B, rightmost molecule). These data are also consistent with the nuclease digestion experiments discussed above.

In conclusion, our structure probing experiments demonstrate that all S/R sequences fold into the 3D structure characteristic of this motif with an exception of one control molecule which is not expected to form the motif. Although exact orientations of all nucleotides cannot be established based on structure probing alone, G24 and U25 are cleaved in a manner consistent with forming the core base triple with A40. The structure probing data validates our observations made during the UV-melting experiments.

### Sequence variation in S/R motifs observed in 16S rRNA sequences from Eubacteria with known optimal growth temperatures

Thus far our analysis was based on the sequence variation data derived from the RNA X-ray structures. These structures come from relatively few organisms and provide limited information on sequence variability. To expand our knowledge of S/R motif sequence variation we analyzed ribosomal sequence alignments. We hypothesize that the organisms living at higher temperatures have more rigid RNA 3D motifs while organisms living at lower temperatures have less rigid structures. In order to test this hypothesis, we constructed a special dataset containing 16S alignments with sequences annotated by the optimal growth temperature and phylogenetic group of the source organism.

#### Finding organisms with known optimal growth temperatures

Bacterial type strains associated with the optimal growth temperature were manually extracted from the literature, with a particular effort devoted to identifying extremophiles. Manual extraction was used because the previous attempt at extracting this data, the PGTdb (43), is outdated and no longer maintained. Species which grow optimally <20°C were classified as psychrophilic; species growing from 20°C to <50°C were classified as mesophilic, species growing between 50°C and less than 70°C were thermophilic while species growing optimally at or >70°C were hyperthermophilic (44). The distribution of organisms in different phylas by their optimal growth temperatures can be found in Supplementary table.

#### Selecting a source of sequence alignments

There are several alternative sources of aligned rRNA sequences. We examine 16S alignments because the 16S is often sequenced and therefore provides a large dataset to examine. Unfortunately, very few 23S sequences for extremophiles are available, so we did not attempt to analyze the large ribosomal subunit. In order to choose one 16S alignment, we aligned two 16S 3D structures from distantly related bacteria *E. coli* (PDB 2AVY) and *T. thermophilus* (PDB: 1J5E) using R3DAlign (45). Next, we compared the resulting structural alignment with sequence alignments downloaded from Green Genes, RDP and SILVA (46–48). As a result, Green Genes alignment has been chosen as it had the most agreement with the R3DAlign structural alignment.

#### Selecting a representative sequence alignment dataset

The representative dataset was created by extracting all sequences corresponding to each species and all of its subspecies with known optimal growth temperatures from the Green Genes 16S alignment updated on October 6, 2010 (http://greengenes.lbl.gov). Each species and its subspecies were represented with a single sequence selected based on the highest A, C, G and U content. This was done in order to reduce the bias due to over representation of some organisms in the dataset. This dataset contains 561 sequences; within these sequences 82 belong to psychrophilic, 328 mesophilic, 108 thermophilic and 44 hyperthermophilic organisms (Supplementary table). We used Uniprot's taxonomy to obtain the phylogenetic information for each organism.

#### Correlating structures with sequence alignments

Since some nucleotides in crystal structures are not resolved, the Green Genes alignment and the sequence from the 3D structure may not match. Therefore, we need to determine the correlation between the columns of the sequence alignments and the structure positions. To this end, we extracted all rows from Green Genes, which came from *E. coli*, and removed all gaps, then aligned each sequence to the sequence from the *E. coli* 3D structure (PDB: 2AVY). The Green Genes sequence with the largest number of aligned columns was used to correlate positions in the Green Genes alignment with the PDB sequence.

#### AG and AA trans Hoogsteen-Sugar base pairs correlate with organism growth temperature

Using the correlations between sequence alignments and 3D structures we now examine the variations in S/R-like motifs H17, H27 and 3WJ found in the 16S rRNA. We extracted the columns from the alignment which corresponded to each motif. We then grouped each sequence by the phylum of its source organism and optimal growth temperature class and then counted unique sequences. The resulting tables can be found in Supplementary Table S1.

**Figure 11.**
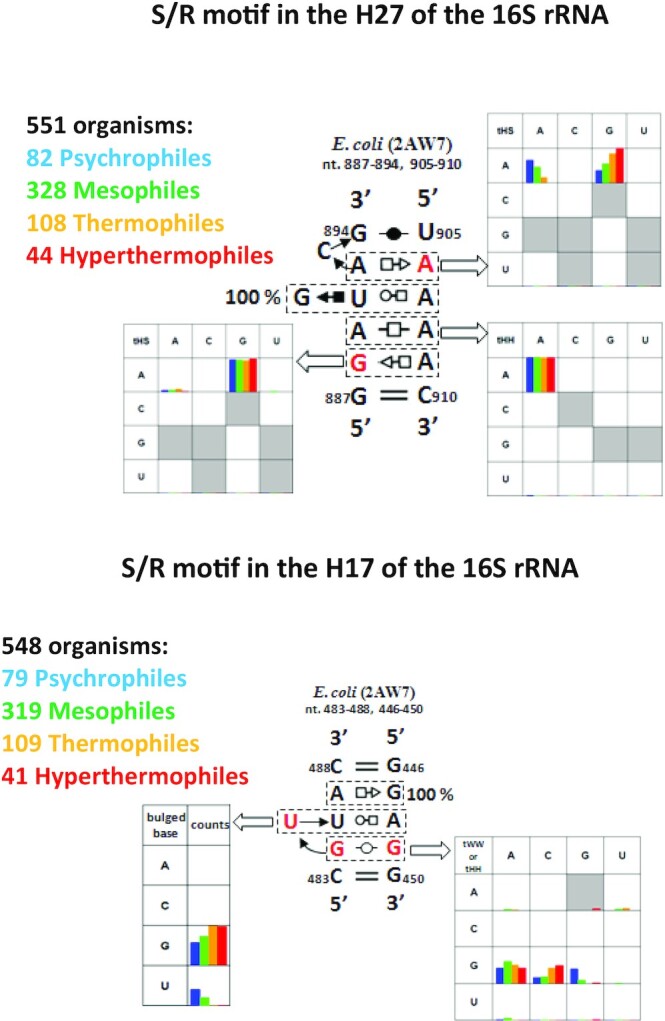
Survey of non-canonical base pairs of S/R motifs in 16S rRNA of Eubacteria. Figure contains 2D diagrams of two S/R motifs one from helix 27 on top and on the bottom is S/R from helix 17. Each bp family has a table with collored bars containing sequence variations of psychrophiles (blue color bar), mesophiles (green color bar), thermophiles (orange color bar) and hyperthermophiles (red color bar). Data in each cell is plotted according to counts of organisms representing sequence of particular non-canonical bp.

Examining the S/R motif in the H27 of the 16S shows a strong correlation between AG and AA in the upper tHS base pair and growth temperature. As temperature increases the fraction of tHS AG also increases (Figure 11 top panel). This does not appear to be due to phylogeny as the variation is found in Bacteriodes, Firmuctues, and Betaproteobacteria. Other positions remain conserved or nearly conserved. This correlation is noticeable only in one tHS base pair of one S/R motif, but it is supported by our observations based on the UV-melting experiments.

The bottom panel in Figure 11 shows the sequence variations of the S/R motif in H17. The bulged base in the triplet is a G in thermophiles and hyperthermophiles, while in psychrophiles it may be a U. All other examined motifs do not show a strong correlation between sequence and optimal growth temperature.

Overall, we do see some variations which may be due to temperature adaptation. However, our current methods are not precise enough to detect subtle changes. Further statistical analysis on a larger dataset is needed to validate these initial, limited findings.

## CONCLUSION

In the present work, we systematically study sequence variation at structurally conserved positions of S/R motif in rRNA from different organisms. Herein, our effort is aimed at gaining insight into the thermodynamics of isosteric and non-isosteric substitutions within the S/R motif. Besides the stability investigation, a structural probing analysis is performed to observe which mutations would disturb the motif.

This study demonstrates that isosteric substitutions do not disturb the S/R motif as it was expected from survey of 3D structures and survey of 16S rRNA from organisms adapted to different temperatures.

The most frequent variation was observed at the ‘lower’ tSH base pair and any neutral substitutions at this place do not significantly impact on the overall stability of the motif. On the other hand, non-isosteric substitutions destabilize the motif but the conformation is appears to be modest as reflected from structural probing experiments.

Substitutions at the ‘upper’ tHS pair are the most crucial for the stability and conformation of the motif. Non-isosteric mutations destroy the geometry of S/R motif. Isosteric mutations tHS A6–A9 or C6–U9 destabilize the motif but the geometry is preserved. Moreover, tHS A6–A9 makes the motif more flexible as reflected by RNAse T1 digestion pattern.

Any mutations at bulged nucleotides participating in triplet interaction significantly impact on the stability of the motif as shown by UV-melting experiments.

Our phylogenetic survey suggests a correlation between non-canonical base pairs and optimal growth temperatures. In high temperature environments *t*HS AG is the most frequent base pair in S/R motifs, whereas tHS AA is prevalent in cold environments. In addition, the bulged G, which is conserved in most organisms, mutates to U, A or C mostly in psychrophiles and mesophiles. This suggests that organisms may adapt to different temperature environments not only by adjusting the number of GC and AU Watson–Crick pairs in the helical regions as demonstrated previously (5), but also by selecting optimal isosteric sequence combinations for their non-canonical base pairs.

Using S/R motif as an example, we have demonstrated that non-canonical interactions in structured RNA motifs are very important to overall structure and stability. By changing base pair at certain non-canonical positions we can control stability, structure, and perhaps function of large RNAs.

## Supplementary Material

gkab703_Supplemental_Files

## References

[1] Stombaugh J., Zirbel C.L., Westhof E., Leontis N.B. Frequency and isostericity of RNA base pairs. Nucleic Acids Res. 2009; 37:2294–2312. 19240142 10.1093/nar/gkp011 PMC2673412

[2] Gutell R.R., Cannone J.J., Shang Z., Du Y., Serra M.J. A story: unpaired adenosine bases in ribosomal RNAs. J. Mol. Biol. 2000; 304:335–354. 11090278 10.1006/jmbi.2000.4172

[3] Gutell R.R., Weiser B., Woese C.R., Noller H.F. Comparative anatomy of 16-S-like ribosomal RNA. Prog. Nucleic Acid Res. Mol. Biol. 1985; 32:155–216. 3911275 10.1016/s0079-6603(08)60348-7

[4] Leontis N.B., Westhof E. Geometric nomenclature and classification of RNA base pairs. RNA. 2001; 7:499–512. 11345429 10.1017/s1355838201002515 PMC1370104

[5] Galtier N., Lobry J.R. Relationships between genomic G+C content, RNA secondary structures, and optimal growth temperature in prokaryotes. J. Mol. Evol. 1997; 44:632–636. 9169555 10.1007/pl00006186

[6] Miralles F. Compositional properties and thermal adaptation of SRP-RNA in bacteria and archaea. J. Mol. Evol. 2010; 70:181–189. 20069286 10.1007/s00239-009-9319-1

[7] Hurst L.D., Merchant A.R. High guanine-cytosine content is not an adaptation to high temperature: a comparative analysis amongst prokaryotes. Proc. Biol. Sci. Roy. Soc. 2001; 268:493–497. 10.1098/rspb.2000.1397 PMC1088632 11296861

[8] Freier S.M., Kierzek R., Jaeger J.A., Sugimoto N., Caruthers M.H., Neilson T., Turner D.H. Improved free-energy parameters for predictions of RNA duplex stability. Proc. Natl. Acad. Sci. U. S. A. 1986; 83:9373–9377. 2432595 10.1073/pnas.83.24.9373 PMC387140

[9] Mathews D.H., Sabina J., Zuker M., Turner D.H. Expanded sequence dependence of thermodynamic parameters improves prediction of RNA secondary structure. J. Mol. Biol. 1999; 288:911–940. 10329189 10.1006/jmbi.1999.2700

[10] Turner D.H., Mathews D.H. NNDB: the nearest neighbor parameter database for predicting stability of nucleic acid secondary structure. Nucleic Acids Res. 2010; 38:D280–D282. 19880381 10.1093/nar/gkp892 PMC2808915

[11] Xia T., SantaLucia J. Jr, Burkard M.E., Kierzek R., Schroeder S.J., Jiao X., Cox C., Turner D.H. Thermodynamic parameters for an expanded nearest-neighbor model for formation of RNA duplexes with Watson–Crick base pairs. Biochemistry. 1998; 37:14719–14735. 9778347 10.1021/bi9809425

[12] Schroeder S.J., Turner D.H. Factors affecting the thermodynamic stability of small asymmetric internal loops in RNA. Biochemistry. 2000; 39:9257–9274. 10924119 10.1021/bi000229r

[13] Lancaster L., Lambert N.J., Maklan E.J., Horan L.H., Noller H.F. The sarcin-ricin loop of 23S rRNA is essential for assembly of the functional core of the 50S ribosomal subunit. RNA. 2008; 14:1999–2012. 18755834 10.1261/rna.1202108 PMC2553751

[14] Shi X., Khade P.K., Sanbonmatsu K.Y., Joseph S. Functional role of the sarcin-ricin loop of the 23S rRNA in the elongation cycle of protein synthesis. J. Mol. Biol. 2012; 419:125–138. 22459262 10.1016/j.jmb.2012.03.016 PMC3348345

[15] Szewczak A.A., Moore P.B. The sarcin/ricin loop, a modular RNA. J. Mol. Biol. 1995; 247:81–98. 7897662 10.1006/jmbi.1994.0124

[16] Leontis N.B., Westhof E. A common motif organizes the structure of multi-helix loops in 16 S and 23 S ribosomal RNAs. J. Mol. Biol. 1998; 283:571–583. 9784367 10.1006/jmbi.1998.2106

[17] Mladek A., Sponer J.E., Kulhanek P., Lu X.J., Olson W.K., Sponer J. Understanding the sequence preference of recurrent RNA building blocks using quantum chemistry: the intrastrand RNA dinucleotide platform. J. Chem. Theory Comput. 2012; 8:335–347. 22712001 10.1021/ct200712b PMC3375708

[18] Leontis N.B., Stombaugh J., Westhof E. Motif prediction in ribosomal RNAs Lessons and prospects for automated motif prediction in homologous RNA molecules. Biochimie. 2002; 84:961–973. 12458088 10.1016/s0300-9084(02)01463-3

[19] Cavaluzzi M.J., Borer P.N. Revised UV extinction coefficients for nucleoside-5′-monophosphates and unpaired DNA and RNA. Nucleic Acids Res. 2004; 32:e13. 14722228 10.1093/nar/gnh015 PMC373314

[20] Borer P.N., Kan L.S., Ts’o P.O. Conformation and interaction of short nucleic acid double-stranded helices. I. Proton magnetic resonance studies on the nonexchangeable protons of ribosyl ApApGpCpUpU. Biochemistry. 1975; 14:4847–4863. 1182124 10.1021/bi00693a012

[21] AbouHaidar M.G., Ivanov I.G. Non-enzymatic RNA hydrolysis promoted by the combined catalytic activity of buffers and magnesium ions. Z Naturforsch C. 1999; 54:542–548. 10488562 10.1515/znc-1999-7-813

[22] McDowell J.A., Turner D.H. Investigation of the structural basis for thermodynamic stabilities of tandem GU mismatches: solution structure of (rGAGGUCUC)2 by two-dimensional NMR and simulated annealing. Biochemistry. 1996; 35:14077–14089. 8916893 10.1021/bi9615710

[23] Borer P.N., Dengler B., Tinoco I. Jr, Uhlenbeck O.C. Stability of ribonucleic acid double-stranded helices. J. Mol. Biol. 1974; 86:843–853. 4427357 10.1016/0022-2836(74)90357-x

[24] Marky L.A., Breslauer K.J. Calculating thermodynamic data for transitions of any molecularity from equilibrium melting curves. Biopolymers. 1987; 26:1601–1620. 3663875 10.1002/bip.360260911

[25] SantaLucia J. Jr, Kierzek R., Turner D.H. Effects of GA mismatches on the structure and thermodynamics of RNA internal loops. Biochemistry. 1990; 29:8813–8819. 2271557 10.1021/bi00489a044

[26] Petrov A.I., Zirbel C.L., Leontis N.B. WebFR3D–a server for finding, aligning and analyzing recurrent RNA 3D motifs. Nucleic Acids Res. 2011; 39:W50–W55. 21515634 10.1093/nar/gkr249 PMC3125732

[27] Sarver M., Zirbel C.L., Stombaugh J., Mokdad A., Leontis N.B. FR3D: finding local and composite recurrent structural motifs in RNA 3D structures. J. Math. Biol. 2008; 56:215–252. 17694311 10.1007/s00285-007-0110-x PMC2837920

[28] Endo Y., Chan Y.L., Lin A., Tsurugi K., Wool I.G. The cytotoxins alpha-sarcin and ricin retain their specificity when tested on a synthetic oligoribonucleotide (35-mer) that mimics a region of 28 S ribosomal ribonucleic acid. J. Biol. Chem. 1988; 263:7917–7920. 3372511

[29] Abu Almakarem A.S., Petrov A.I., Stombaugh J., Zirbel C.L., Leontis N.B. Comprehensive survey and geometric classification of base triples in RNA structures. Nucleic Acids Res. 2012; 40:1407–1423. 22053086 10.1093/nar/gkr810 PMC3287178

[30] Alam S., Grum-Tokars V., Krucinska J., Kundracik M.L., Wedekind J.E. Conformational heterogeneity at position U37 of an all-RNA hairpin ribozyme with implications for metal binding and the catalytic structure of the S-turn. Biochemistry. 2005; 44:14396–14408. 16262240 10.1021/bi051550i

[31] Greenbaum N.L., Mundoma C., Peterman D.R. Probing of metal-binding domains of RNA hairpin loops by laser-induced lanthanide(III) luminescence. Biochemistry. 2001; 40:1124–1134. 11170437 10.1021/bi002210u

[32] Serra M.J., Baird J.D., Dale T., Fey B.L., Retatagos K., Westhof E. Effects of magnesium ions on the stabilization of RNA oligomers of defined structures. RNA. 2002; 8:307–323. 12003491 10.1017/s1355838202024226 PMC1370253

[33] Hermann T., Westhof E. Exploration of metal ion binding sites in RNA folds by Brownian-dynamics simulations. Structure. 1998; 6:1303–1314. 9782053 10.1016/s0969-2126(98)00130-0

[34] Sponer J.E., Reblova K., Mokdad A., Sychrovsky V., Leszczynski J., Sponer J. Leading RNA tertiary interactions: structures, energies, and water insertion of A-minor and P-interactions. A quantum chemical view. J. Phys. Chem. B. 2007; 111:9153–9164. 17602515 10.1021/jp0704261

[35] Mladek A., Sharma P., Mitra A., Bhattacharyya D., Sponer J., Sponer J.E. Trans Hoogsteen/sugar edge base pairing in RNA. Structures, energies, and stabilities from quantum chemical calculations. J. Phys. Chem. B. 2009; 113:1743–1755. 19152254 10.1021/jp808357m

[36] Zgarbova M., Jurecka P., Banas P., Otyepka M., Sponer J.E., Leontis N.B., Zirbel C.L., Sponer J. Noncanonical hydrogen bonding in nucleic acids. Benchmark evaluation of key base-phosphate interactions in folded RNA molecules using quantum-chemical calculations and molecular dynamics simulations. J. Phys. Chem. A. 2011; 115:11277–11292. 21910417 10.1021/jp204820b

[37] Zirbel C.L., Sponer J.E., Sponer J., Stombaugh J., Leontis N.B. Classification and energetics of the base-phosphate interactions in RNA. Nucleic Acids Res. 2009; 37:4898–4918. 19528080 10.1093/nar/gkp468 PMC2731888

[38] Seggerson K., Moore P.B. Structure and stability of variants of the sarcin-ricin loop of 28S rRNA: NMR studies of the prokaryotic SRL and a functional mutant. RNA. 1998; 4:1203–1215. 9769095 10.1017/s1355838298980773 PMC1369693

[39] Correll C.C., Swinger K. Common and distinctive features of GNRA tetraloops based on a GUAA tetraloop structure at 1.4 A resolution. RNA. 2003; 9:355–363. 12592009 10.1261/rna.2147803 PMC1370402

[40] Sorin E.J., Engelhardt M.A., Herschlag D., Pande V.S. RNA simulations: probing hairpin unfolding and the dynamics of a GNRA tetraloop. J. Mol. Biol. 2002; 317:493–506. 11955005 10.1006/jmbi.2002.5447

[41] Andersen J., Delihas N., Hanas J.S., Wu C.W. 5S RNA structure and interaction with transcription factor A. 1. Ribonuclease probe of the structure of 5S RNA from Xenopus laevis oocytes. Biochemistry. 1984; 23:5752–5759. 6084515 10.1021/bi00319a013

[42] Chan Y.L., Wool I.G. The integrity of the sarcin/ricin domain of 23 S ribosomal RNA is not required for elongation factor-independent peptide synthesis. J. Mol. Biol. 2008; 378:12–19. 18342885 10.1016/j.jmb.2008.02.016

[43] Huang S.L., Wu L.C., Liang H.K., Pan K.T., Horng J.T., Ko M.T. PGTdb: a database providing growth temperatures of prokaryotes. Bioinformatics. 2004; 20:276–278. 14734322 10.1093/bioinformatics/btg403

[44] Rothschild L.J., Mancinelli R.L. Life in extreme environments. Nature. 2001; 409:1092–1101. 11234023 10.1038/35059215

[45] Rahrig R.R., Leontis N.B., Zirbel C.L. R3D Align: global pairwise alignment of RNA 3D structures using local superpositions. Bioinformatics. 2010; 26:2689–2697. 20929913 10.1093/bioinformatics/btq506 PMC3465099

[46] Cole J.R., Wang Q., Cardenas E., Fish J., Chai B., Farris R.J., Kulam-Syed-Mohideen A.S., McGarrell D.M., Marsh T., Garrity G.M. et al. The Ribosomal Database Project: improved alignments and new tools for rRNA analysis. Nucleic Acids Res. 2009; 37:D141–D145. 19004872 10.1093/nar/gkn879 PMC2686447

[47] DeSantis T.Z., Hugenholtz P., Larsen N., Rojas M., Brodie E.L., Keller K., Huber T., Dalevi D., Hu P., Andersen G.L. Greengenes, a chimera-checked 16S rRNA gene database and workbench compatible with ARB. Appl. Environ. Microbiol. 2006; 72:5069–5072. 16820507 10.1128/AEM.03006-05 PMC1489311

[48] Pruesse E., Quast C., Knittel K., Fuchs B.M., Ludwig W., Peplies J., Glockner F.O. SILVA: a comprehensive online resource for quality checked and aligned ribosomal RNA sequence data compatible with ARB. Nucleic Acids Res. 2007; 35:7188–7196. 17947321 10.1093/nar/gkm864 PMC2175337

[49] Klein D.J., Moore P.B., Steitz T.A. The roles of ribosomal proteins in the structure assembly, and evolution of the large ribosomal subunit. J. Mol. Biol. 2004; 340:141–177. 15184028 10.1016/j.jmb.2004.03.076

[50] Schuwirth B.S., Borovinskaya M.A., Hau C.W., Zhang W., Vila-Sanjurjo A., Holton J.M., Cate J.H. Structures of the bacterial ribosome at 3.5 A resolution. Science. 2005; 310:827–834. 16272117 10.1126/science.1117230

[51] Bulkley D., Johnson F., Steitz T.A. The antibiotic thermorubin inhibits protein synthesis by binding to inter-subunit bridge B2a of the ribosome. J. Mol. Biol. 2012; 416:571–578. 22240456 10.1016/j.jmb.2011.12.055 PMC3336878

[52] Harms J.M., Wilson D.N., Schluenzen F., Connell S.R., Stachelhaus T., Zaborowska Z., Spahn C.M., Fucini P. Translational regulation via L11: molecular switches on the ribosome turned on and off by thiostrepton and micrococcin. Mol. Cell. 2008; 30:26–38. 18406324 10.1016/j.molcel.2008.01.009

[53] Krasilnikov A.S., Yang X., Pan T., Mondragon A. Crystal structure of the specificity domain of ribonuclease P. Nature. 2003; 421:760–764. 12610630 10.1038/nature01386

[54] Serganov A., Huang L., Patel D.J. Structural insights into amino acid binding and gene control by a lysine riboswitch. Nature. 2008; 455:1263–1267. 18784651 10.1038/nature07326 PMC3726722

[55] Klinge S., Voigts-Hoffmann F., Leibundgut M., Arpagaus S., Ban N. Crystal structure of the eukaryotic 60S ribosomal subunit in complex with initiation factor 6. Science. 2011; 334:941–948. 22052974 10.1126/science.1211204

[56] Ben-Shem A., Garreau de Loubresse N., Melnikov S., Jenner L., Yusupova G., Yusupov M. The structure of the eukaryotic ribosome at 3.0 A resolution. Science. 2011; 334:1524–1529. 22096102 10.1126/science.1212642

[57] Lu D., Searles M.A., Klug A. Crystal structure of a zinc-finger-RNA complex reveals two modes of molecular recognition. Nature. 2003; 426:96–100. 14603324 10.1038/nature02088

[58] Correll C.C., Beneken J., Plantinga M.J., Lubbers M., Chan Y.L. The common and the distinctive features of the bulged-G motif based on a 1.04 A resolution RNA structure. Nucleic Acids Res. 2003; 31:6806–6818. 14627814 10.1093/nar/gkg908 PMC290275

[59] Leontis N.B., Stombaugh J., Westhof E. The non-Watson–Crick base pairs and their associated isostericity matrices. Nucleic Acids Res. 2002; 30:3497–3531. 12177293 10.1093/nar/gkf481 PMC134247

