# Supplementary material for: Context-sensitivity of isosteric substitutions of non-Watson–Crick basepairs in recurrent RNA 3D motifs
Source: Nucleic Acids Res. 2021 Aug 17;49(16):9574–93. doi: 10.1093/nar/gkab703 (PMC8450098; doi:10.1093/nar/gkab703)
Supplement: gkab703_Supplemental_Files [file gkab703_supplemental_files.zip › Revised_Supplementary figures.docx]

**Context-sensitivity of Isosteric Substitutions of non-Watson-Crick Basepairs in Recurrent RNA 3D Motifs**

Emil F. Khisamutdinov^1,2*^, Blake A. Sweeney^3,4^ and Neocles B. Leontis^1^.

^1^ Department of Chemistry and Center for Photochemical Science, Bowling Green State University, Bowling Green, OH 43403, USA

^2^ Department of Chemistry, Ball State University, Muncie, IN 47306, USA

^3^ Department of Biological Sciences, Bowling Green State University, Bowling Green, OH 43403, USA

^4^ European Molecular Biology Laboratory, European Bioinformatics Institute, Cambridge, UK.

* To whom correspondence should be addressed. Tel: 765-285-8084; Fax: 765-285-6505; Email: [kemil@bsu.edu](mailto:kemil@bsu.edu)

**1. Supplementary Figures**

**Figure S1.** Isosteric and near isosteric relationships between basepairs frequently observed in S/R motif. Structures shown are actual instances from experimental data obtained from PDB, representing exemplars of all annotated base pairs of the given base combination and geometric family ([1](#_ENREF_1)). A) *trans-*Hoogsteen/sugar edge base pair combinations. B) *trans*-Hoogsteen/ Hoogsteen base pair combinations.


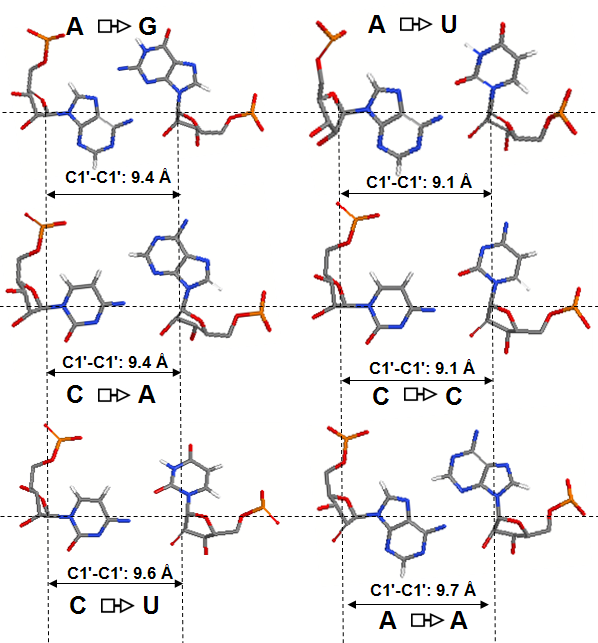

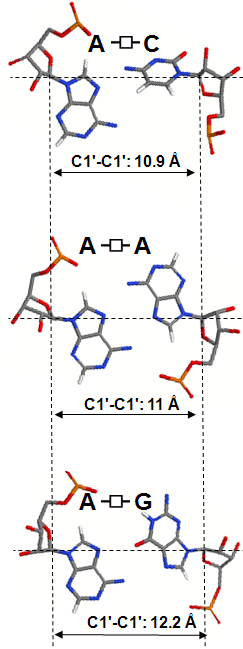


**A**

**B**

**Figure S2.** Determination of thermodynamic parameters for U2G/C12A duplexes. Optical melting experiments were performed in 10 mM MgCl_2_, 100 mM NaCl, 10 mM sodium cacodylate and 0.5 mM Na_2_EDTA (pH 6.9). Melting curves (absorbance vs temperature) were obtained using a heating rate of 1° C/min from 15 to 90 °C on a Cary Bio-100 spectrometer with a temperature controller. The absorbance was measured at 260 nm. *Meltwin* ([2](#_ENREF_2)) was used to fit melting curves to a two state model, assuming linear sloping baselines and temperature independent Δ*H*° and Δ*S*° values. T_M_ values at different (5, 10, 15 and 20 μM ) concentrations were used to calculate thermodynamic parameters of non-self-complementary duplexes using Van’t Hoff plot of T_M_ ^-1^ versus log (C_T_/4).

**
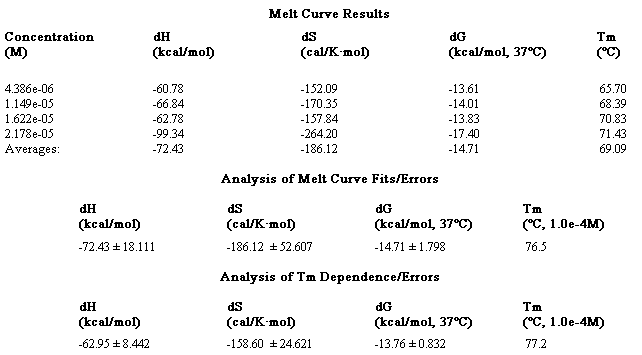
**

**
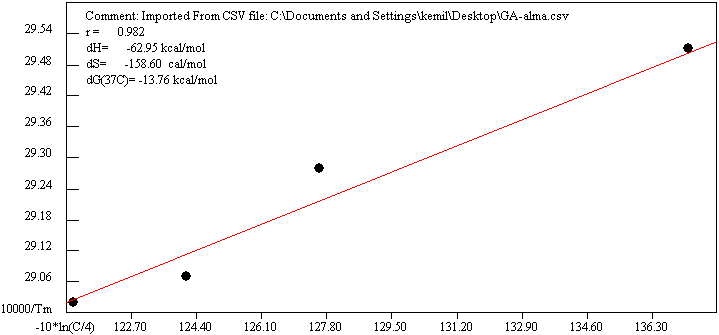
**

**References**

1. Stombaugh, J., Zirbel, C.L., Westhof, E. and Leontis, N.B. (2009) Frequency and isostericity of RNA base pairs. *Nucleic acids research*, **37**, 2294-2312.

2. McDowell, J.A. and Turner, D.H. (1996) Investigation of the structural basis for thermodynamic stabilities of tandem GU mismatches: solution structure of (rGAGGUCUC)2 by two-dimensional NMR and simulated annealing. *Biochemistry*, **35**, 14077-14089.
